# Unity in diversity—food plants and fungi of Sakartvelo (Republic of Georgia), Caucasus

**DOI:** 10.1186/s13002-021-00490-9

**Published:** 2021-12-31

**Authors:** Rainer W. Bussmann, Narel Y. Paniagua Zambrana, Inayat Ur Rahman, Zaal Kikvidze, Shalva Sikharulidze, David Kikodze, David Tchelidze, Manana Khutsishvili, Ketevan Batsatsashvili

**Affiliations:** 1 Department of Ethnobotany, Institute of Botany and Bakuriani Alpine Botanical Garden, Ilia State University, Botanikuri St. 1, 0105 Tbilisi, Georgia; 2 Herbario Nacional de Bolivia, Instituto de Ecología-UMSA, Campus Universitario, Cota Cota Calle 27, La Paz, Bolivia; 3 Department of Botany, Hazara University, Mansehra, 21300 KP Pakistan

**Keywords:** Republic of Georgia, Caucasus, Traditional Knowledge, Knowledge loss, Food plants, Conservation

## Abstract

**Background:**

The Republic of Georgia is part of the Caucasus biodiversity hotspot, and human agricultural plant use dates back at least 6000 years. Over the last years, lots of ethnobotanical research on the area has been published. In this paper, we analyze the use of food plants in the 80% of Georgia not occupied by Russian forces. We hypothesized that (1) given the long tradition of plant use, and the isolation under Soviet rule, plant use both based on home gardens and wild harvesting would be more pronounced in Georgia than in the wider region, (2) food plant use knowledge would be widely and equally spread in most of Georgia, (3) there would still be incidence of knowledge loss despite wide plant use, especially in climatically favored agricultural regions in Western and Eastern Georgia.

**Methods:**

From 2013 to 2019, we interviewed over 380 participants in all regions of Georgia not occupied by Russian forces and recorded over 19,800 mentions of food plants. All interviews were carried out in the participants’ homes and gardens by native speakers of Georgian and its dialects (Imeretian, Rachian, Lechkhumian, Tush, Khevsurian, Psavian, Kakhetian), other Kartvelian languages (Megrelian, Svan) and minority languages (Ossetian, Ude, Azeri, Armenian, Greek).

**Results:**

The regional division was based primarily on historic provinces of Georgia, which often coincides with the current administrative borders. The total number of taxa, mostly identified to species, including their varieties, was 527. Taxonomically, the difference between two food plant groups—garden versus wild—was strongly pronounced even at family level. The richness of plant families was 65 versus 97 families in garden versus wild plants, respectively, and the difference was highly significant. Other diversity indices also unequivocally pointed to considerably more diverse family composition of wild collected versus garden plants as the differences between all the tested diversity indices appeared to be highly significant.

The wide use of leaves for herb pies and lactofermented is of particular interest. Some of the ingredients are toxic in larger quantities, and the participants pointed out that careful preparation was needed. The authors explicitly decided to not give any recipes, given that many of the species are widespread, and compound composition—and with it possible toxic effects—might vary across the distribution range, so that a preparation method that sufficiently reduces toxicity in the Caucasus might not necessary be applicable in other areas.

**Conclusions:**

Relationships among the regions in the case of wild food plants show a different and clearer pattern. Adjacent regions cluster together (Kvemo Zemo Racha, and Zemo Imereti; Samegrelo, Guria, Adjara, Lechkhumi and Kvemo and Zemo Svaneti; Meskheti, Javakheti, Kvemo Kartli; Mtianeti, Kakheti, Khevsureti, Tusheti. Like in the case of the garden food plants, species diversity of wild food plants mentioned varied strongly. Climate severity and traditions of the use of wild food plants might play role in this variation. Overall food plant knowledge is widely spread all-across Georgia, and broadly maintained.

## Background

**Fig. 1:**
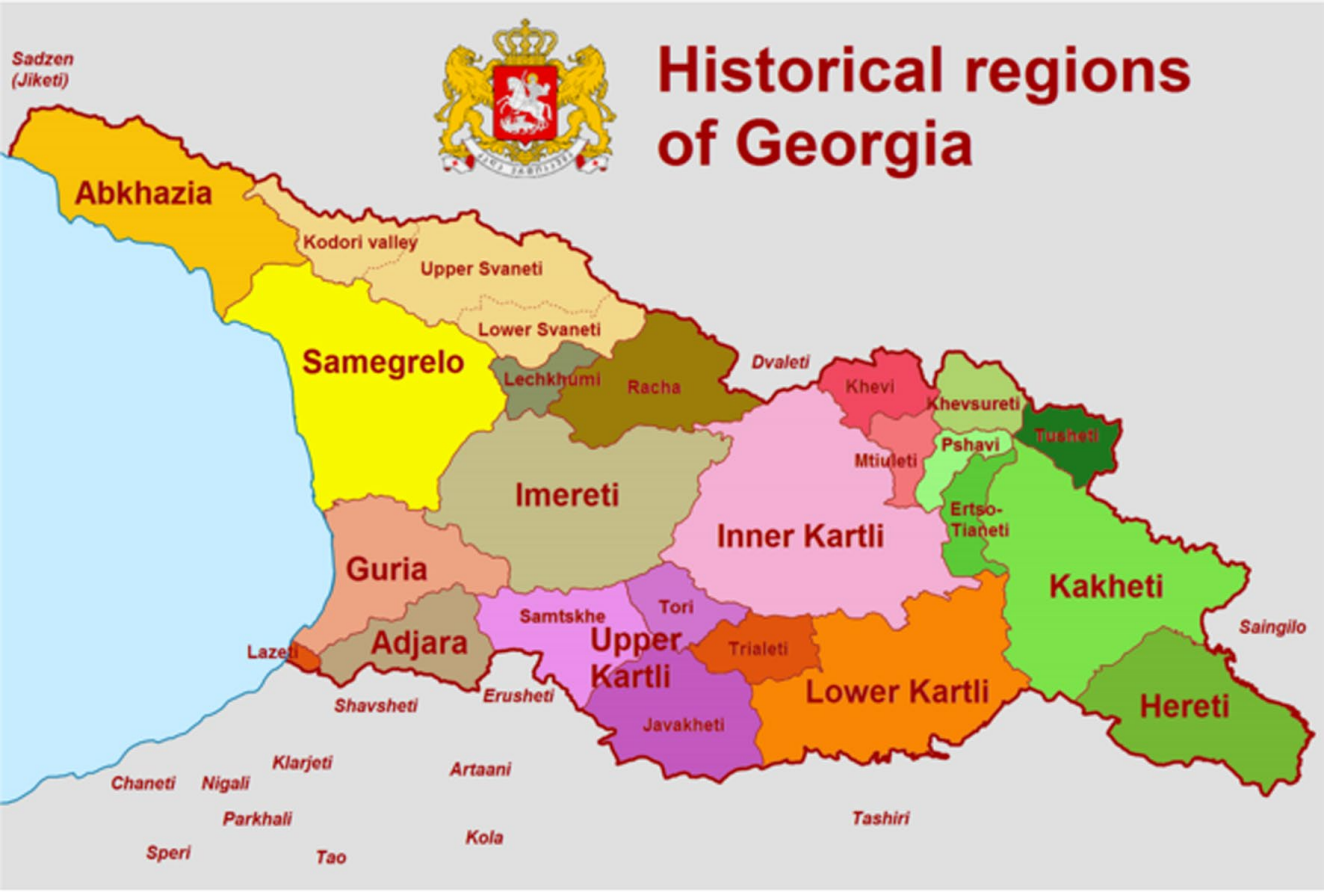
Location and historical provinces of Georgia

Georgia is situated between latitudes 41° and 44° N, and longitudes 40° and 47° E, with an area of 69,700 km^2^, with 20% of the country currently occupied by Russian forces (Fig. 1). Georgia politically associates with European Union and takes part in all major programs of European development and cooperation. Georgia can be defined as a transcontinental country on the divide between Asia and Europe, with its larger part located south to this divide (i.e., in Asia) and smaller but strategically important parts (Khevi, Piriketi Khevsureti, etc.) located north of the continent divide (i.e., in Europe) [1].

The uplift of the Georgian Caucasus started in the late Oligocene and shares the same structural characteristics as the younger mountains of Europe. The Greater Caucasus mostly includes Cretaceous and Jurassic rocks, interspersed with Paleozoic and Precambrian formations in higher regions. Hard, crystalline, metamorphosed rocks like schist and gneisses, as well as pre-Jurassic granites are found in the western part, while softer, Early and Middle Jurassic clayey schist and sandstones in the eastern part. The foot of the Greater Caucasus are built of younger limestone, sandstones, and marls. The Lesser Caucasus in contrast is predominantly formed of Paleogene rocks interspersed with Jurassic and Cretaceous formations. The youngest geological structures of Georgia are represented by the vast volcanic plateaus in the southern part of country. These divisions lead to an extremely complex terrain with pronounced climatic gradients: (1) the mountains of the greater Caucasus with peaks over 5000 m (Shkara, Babis Mta, Chanchakhi, etc.); (2) the inter-mountain plains between the Greater and Lesser Caucasus mountains; (3) the mountains of the Lesser Caucasus with peaks rarely exceeding 3000 m (Mepistskaro, Kheva, Shavi Klde, Kanis Mta, Arsiani); (4) the Volcanic plateau of the Southern Georgia with elevations from 1300 to 2200 m [2–4].

Georgia’s climate is influenced by its location in the warm temperate zone stretching from the Black to the Caspian Seas, and the complexity of its terrain. Georgia has a coastline of 330 km with warm climate, the mean temperature reaching 4–7 °C in January and 22–23 °C in July, and high precipitation (1500–2000 mm annually). The warm oceanic-subtropical climate can be found only at lower elevations (less than 650 m); in more elevated terrains and to the north and east the climate becomes moderately warm. The Greater Caucasus bars cold air from the north, while warm and moist air from the Black Sea spreads easily into the coastal lowlands until reaching the Likhi range, which partly impedes further westward movement of the warm and moist air. In central Georgia, precipitation in mountains can be twice that in the plains. In the mountains, weather conditions change to cool and wet quite steeply with increasing elevation and above 2100 m the environment becomes sub-alpine and alpine, with permanent snow and ice above 3600 m [2–4].

### Plant use history

The Caucasus is regarded as global biodiversity hotspot [5–8]. Botanical has a long history, and the vegetation composition as well as flora are well-known [2, 3].

The territory of modern-day Georgia (Fig. 1) has been inhabited since the early Stone Age, and agriculture was already well-developed during the early Neolithic [9], although human occupation started already in the Early Pleistocene, with the 1.7-Myr-old hominid fossils of Dmanisi in Southern Georgia being the earliest known hominid-site outside of Africa [10–12]. The history of plant and animal use has been documented since the Upper Paleolithic through fossils found in Dzudzuana Cave, dated to ~ 36–34 Ka BP, including wool (*Capra caucasica*), and dyed fibers of wild flax (*Linum usitatissimum*) [13]. Archeological findings from the Neolithic and Early Bronze periods dating back to the 6th–2nd millennium BC are rich with plant fossils and seeds of both wild species and local landraces [14]. The earliest seeds of *Vitis vinifera* (grapevine) were excavated in southern Georgia and date to about 8000 years BP [15]. Medicinal species like *Alchemilla millefolium*, *Artemisia annua*, *A. absinthium*, *Centaurea jacea* and *Urtica dioica* found in the archeological record are still used in the modern pharmacopoeia [16].

Due to its ancient roots agriculture in Georgia is characterized by a great diversity of landraces, and endemic species of crops, already documented in Soviet times [17–22]. However, starting with the implementation of Stalinist agricultural reforms in the 1950s, a rapid loss of local cultivars occurred [23–26]. This process accelerated during post-independence, and knowledge loss has been shown to even extent to aggravate wolf-human conflicts [27]. However, a wide variety of local cultivars can still be found in case of *Vitis vinifera* (Vitaceae) shows its highest genetic diversity in Georgia, with over 600 varieties known, and several dozen used commercially [9, 15, 28–31]. In contrast, essentially none of the 144 varieties, and 150 forms of wheat (*Triticum*) registered in Georgia in the 1940s [21, 22] are sown in modern Georgian commercial agriculture [25], although traditional varieties are still reported from nearby Turkey [32]. The situation is similar in case of *Hordeum vulgare* (Poaceae) which originally was important in beer production, for religious rituals and traditional medicine [9, 33] and *Secale cereale* (Poaceae) [34].

In contrast to the loss of cereals, legumes like peas (*Pisum sativum*), lentils (*Lens cornicularis*), chickpeas (*Cicer arietinum*), fava beans (*Vicia faba*), and vegetables like garden lettuce (*Lactuca sativa*), beans (*Phaseolus vulgaris*), basil (*Ocimum basilicum*), peppermint (*Mentha* x *piperita*), onions (*Allium cepa*), sugar beets (*Beta vulgaris*), spinach (*Spinacia oleracea*), carrots (*Daucus carota*), radishes (*Raphanus sativus*), turnips (*Brassica rapa* var. *rapa*), welsh onion (*Allium fistulosum*), amaranth (*Amaranthus viridis*), goosefoot (*Chenopodium album*), leeks (*Allium ampeloprasum*) and garlic (*Allium sativum*) are still common in home gardens. Herbs like parsley (*Petroselinum crispum*), coriander (*Coriandrum sativum*), tarragon (*Artemisia dracunculus*), savory (*Satureja hortensis*), garden cress (*Lepidium sativum*), dill (*Anethum graveolens*), fennel (*Foeniculum vulgare*), celery (*Apium dulce*), and *Allium fistulosum* (Chinese onion are widely cultivated and popular ingredients of local cuisine [1]. The maintenance of such diversity is of high importance as source material for global crop production [35, 36]. Many species are widely sold as medicines [37].

Over the last years, ethnobotanical research in Georgia has received a large boost, and a wide variety of studies on all aspects of plant use have been published [38–52]. Few of these however focused entirely of food plants [38, 52], many of which are still cultivated in small home-gardens. Home-gardens are often cited as important reservoirs for crop germplasm [53–58] and are mostly sources of food [59, 60]. In wider Eurasia, home gardens have been shown to be an important repository of plant diversity are often part of complex seed exchange networks [61–64].

Given the trajectory of ethnobotanical studies in Georgia, a meta-analysis of the data food plant uses was long overdue. In this publication, we hypothesized that (1) given the long tradition of plant use, and the isolation under Soviet rule, plant use both based on home gardens and wild harvesting would be more pronounced in Georgia than in the wider region, (2) food plant use knowledge would be widely and equally spread in most of Georgia, (3) there would still be incidence of knowledge loss despite wide plant use, especially in climatically favored agricultural regions in Western and Eastern Georgia.

## Materials and methods

### Ethnobotanical interviews

From 2013 to 2019, we interviewed over 380 participants in all regions of Georgia not occupied by Russian forces on their general plant use, recording over 32,000 individual uses. The analyses of all uses have been published in a variety of papers [41–50]. However, of all uses over 19,800 mentions were of food plants, which is why we regarded it as prudent to present a separate analysis of these. Interviews using semi-structured questionnaires were conducted after obtaining the oral prior informed consent of the participants, which were selected by snowball sampling, trying to reach gender balance and representing different age groups. Most participants were however over 50 years old, as interviews targeted remote villages where only very few younger people remain. All interviews were carried out in the participants’ homes and gardens by native speakers of Georgian and its dialects (Imeretian, Rachian, Lechkhumian, Tush, Khevsurian, Psavian, Kakhetian), other Kartvelian languages (Megrelian, Svan) and minority languages (Ossetian, Ude, Azeri, Armenian, Greek). The languages in which a plant was mentioned are indicated in Table 1. Interviews were subsequently translated into English. Plants grown in home gardens were used as prompts, while wild-collected species were free listed. We classified species as "garden" when they were grown/collected in cultivated areas, and as "forest/wild-collected" when growing and harvested in the wild. We maintained the distinction of "forest" and "garden" because it was used in our previous publications from the region [50], to maintain consistency. In contrast to many other countries Georgia benefits from a complete flora [65–69] and a broad inventory of vernacular names in all languages [68]. Species were identified directly in the field, using this literature, and vouchers collected and deposited in the National Herbarium of Georgia (TBI). The nomenclature of all species follows www.tropicos.org, under APGIII [70]. Collection permits were provided through the Institute of Botany, Ilia State University, Tbilisi.

**Table 1:**
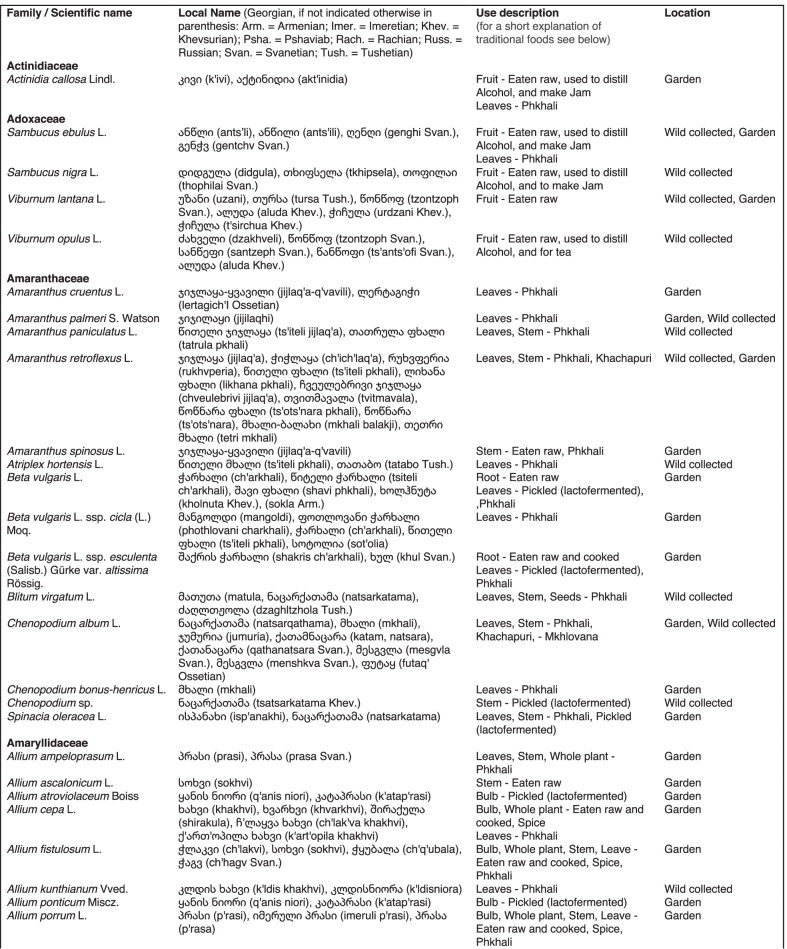

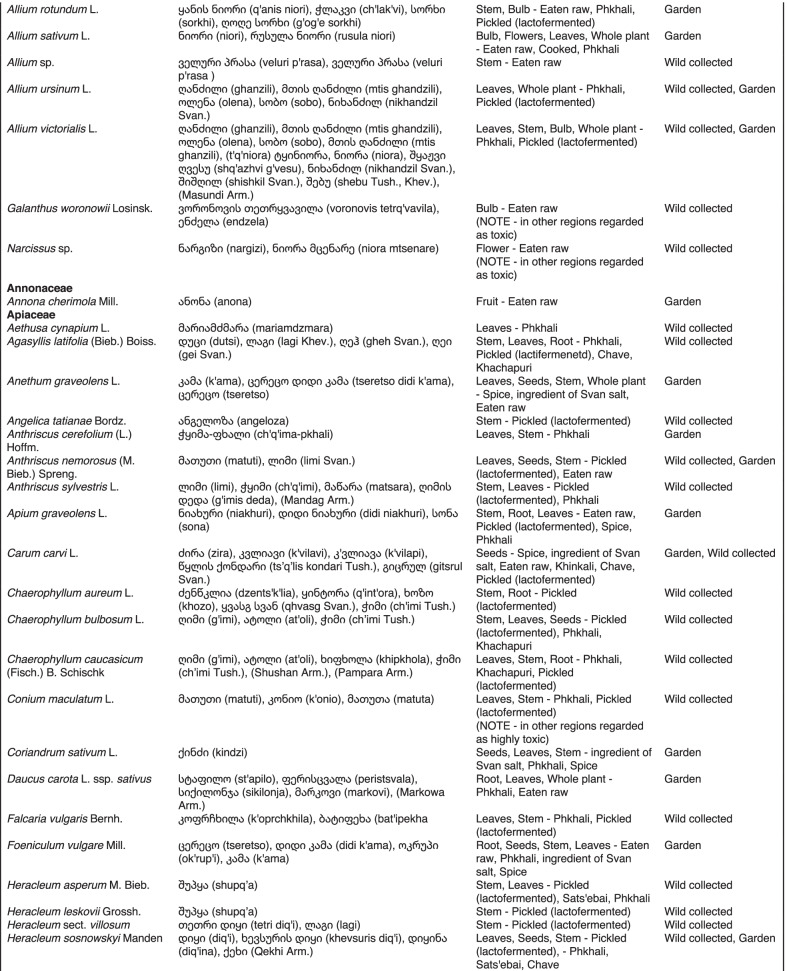

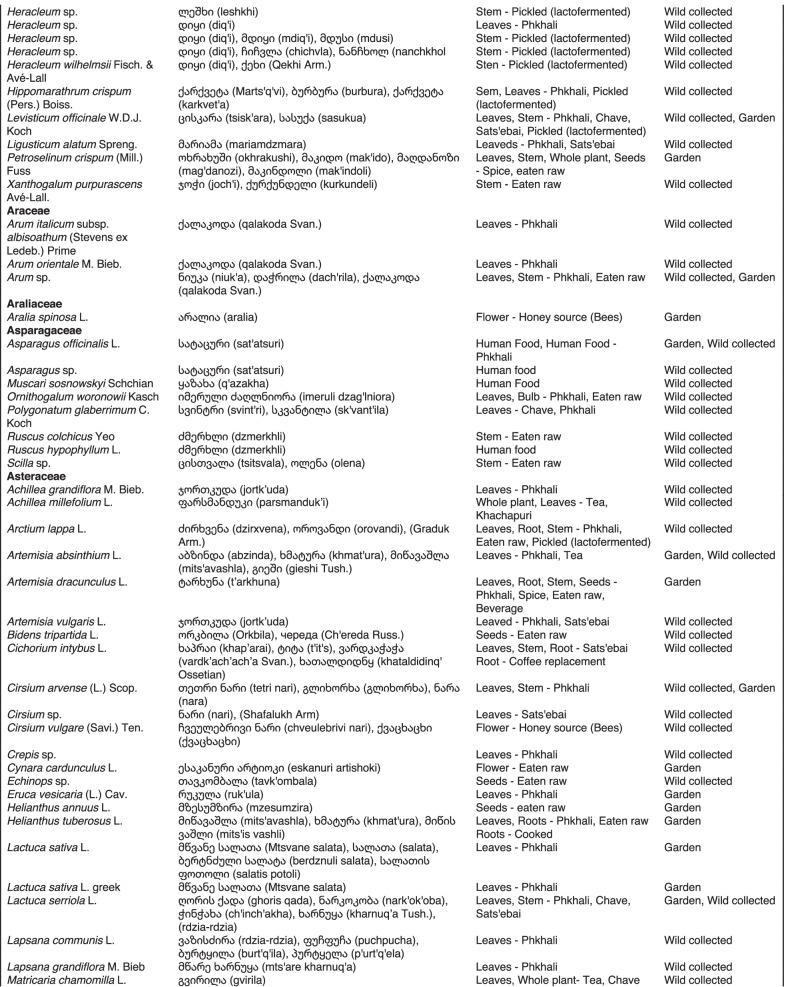

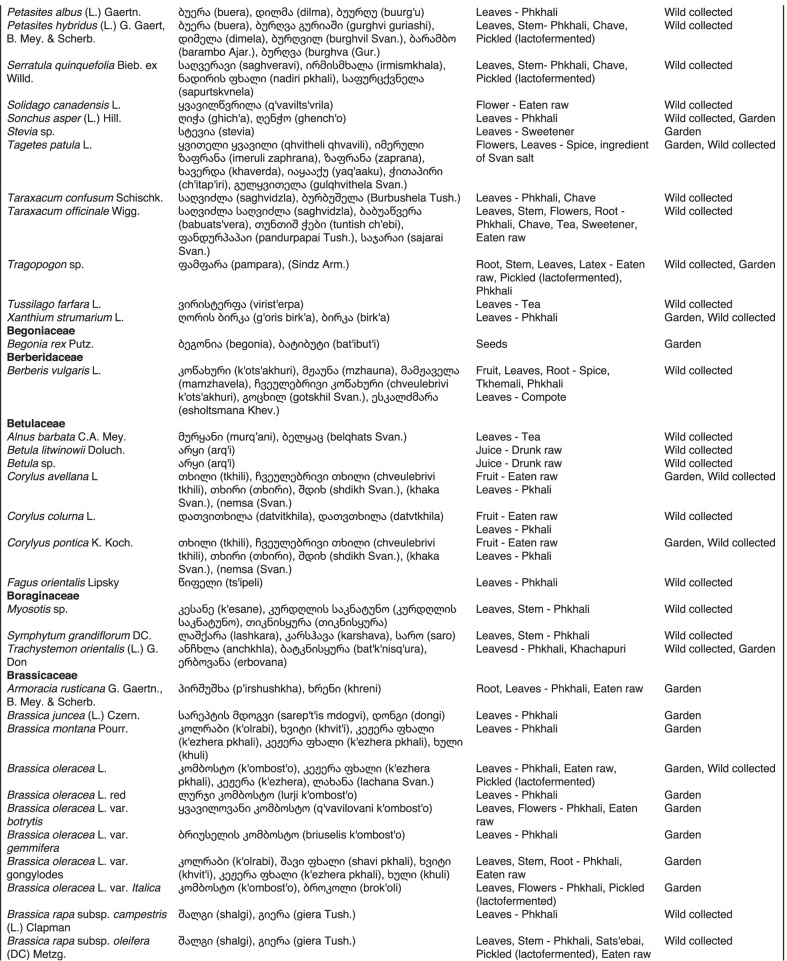

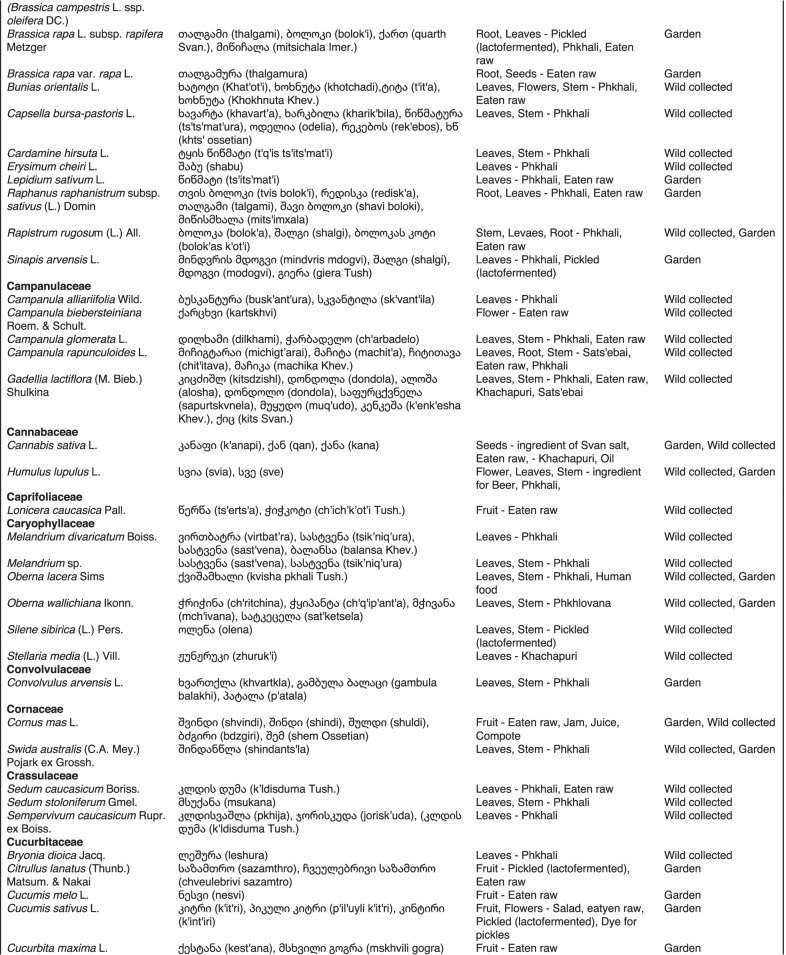

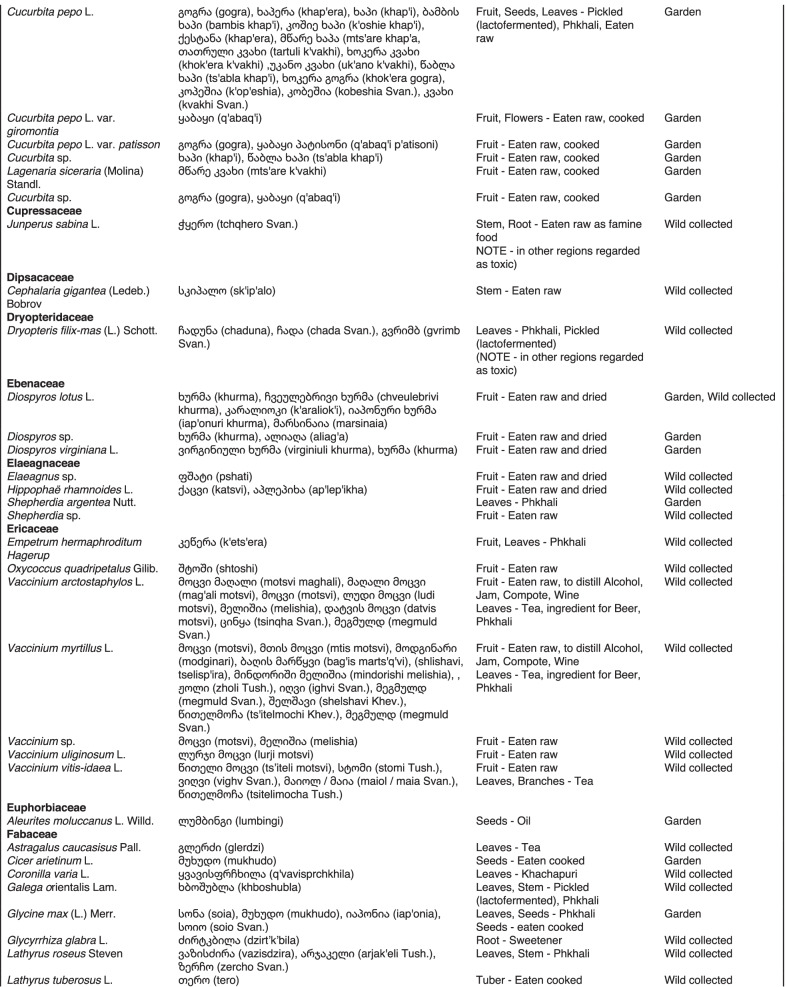

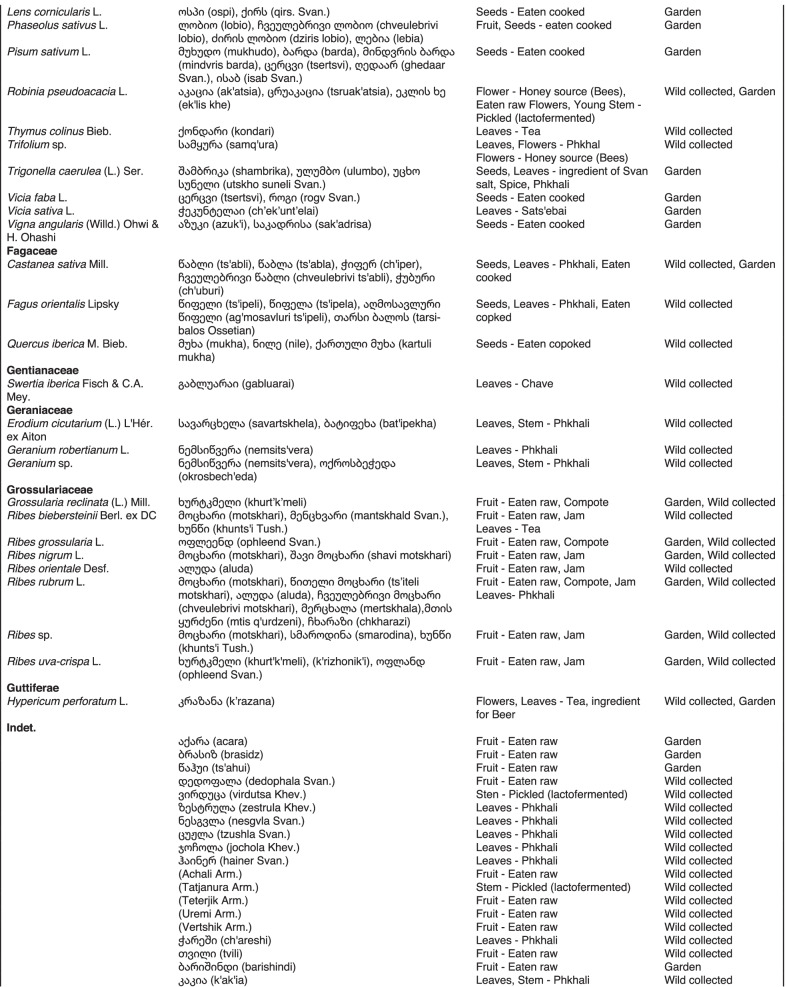

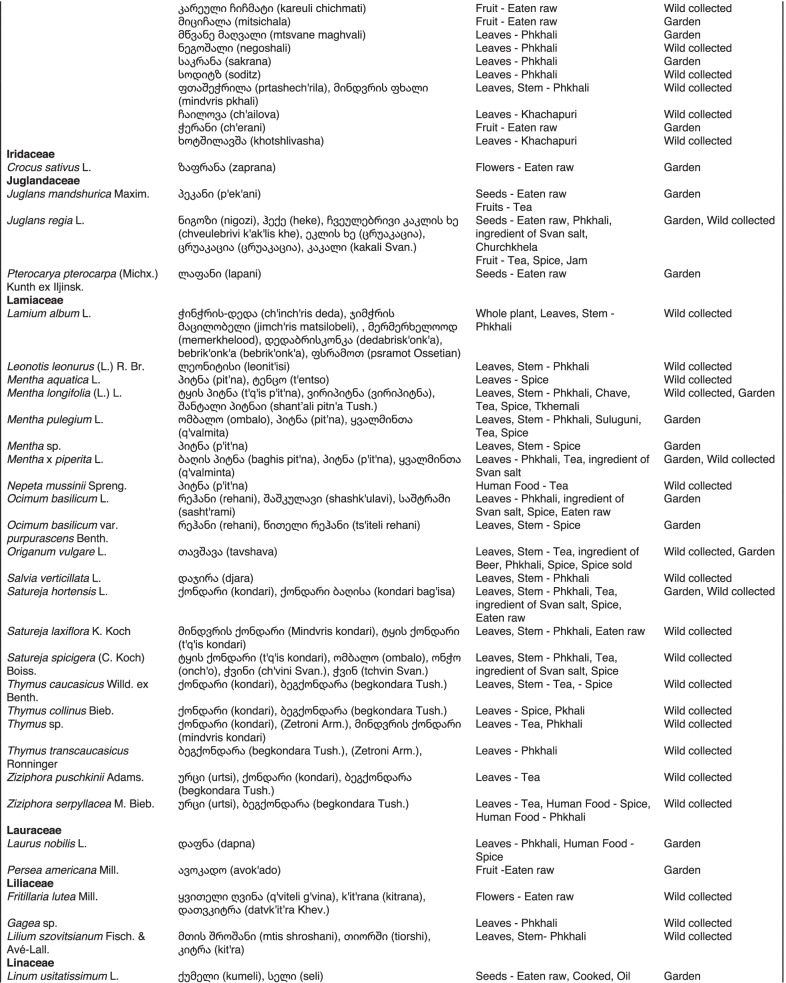

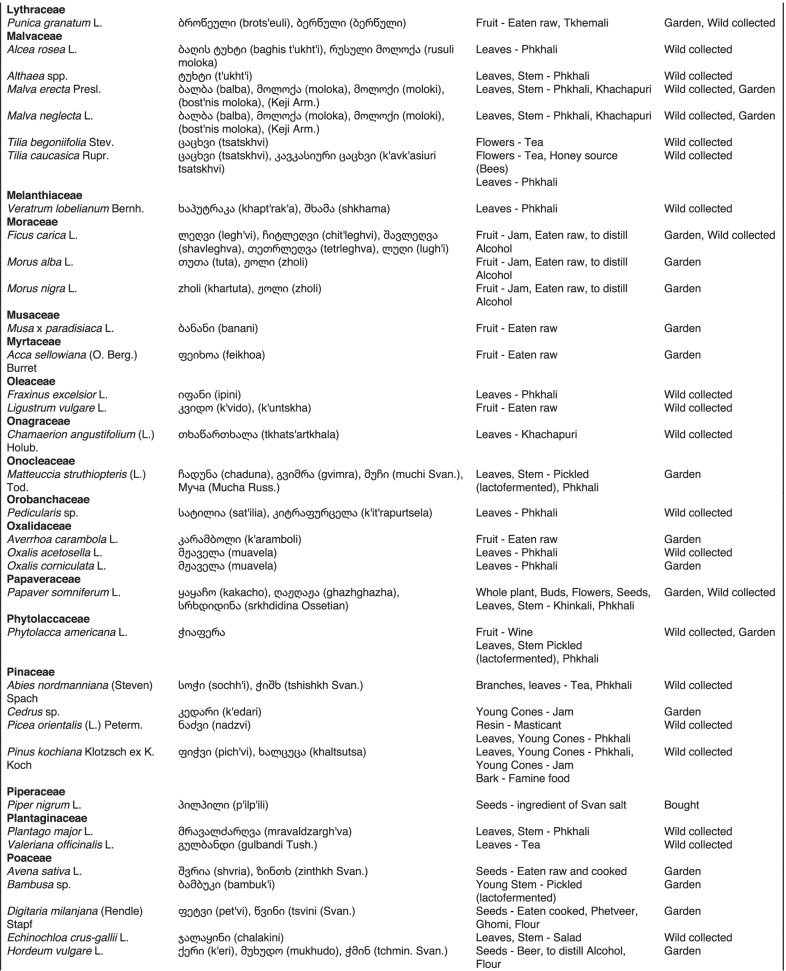

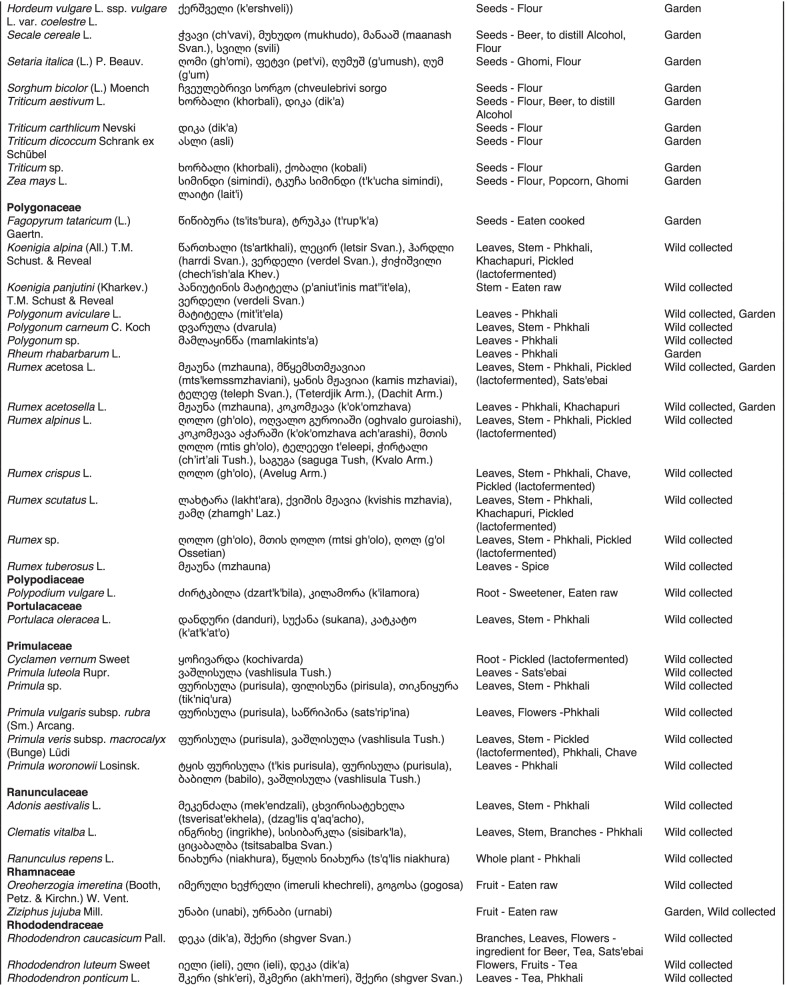

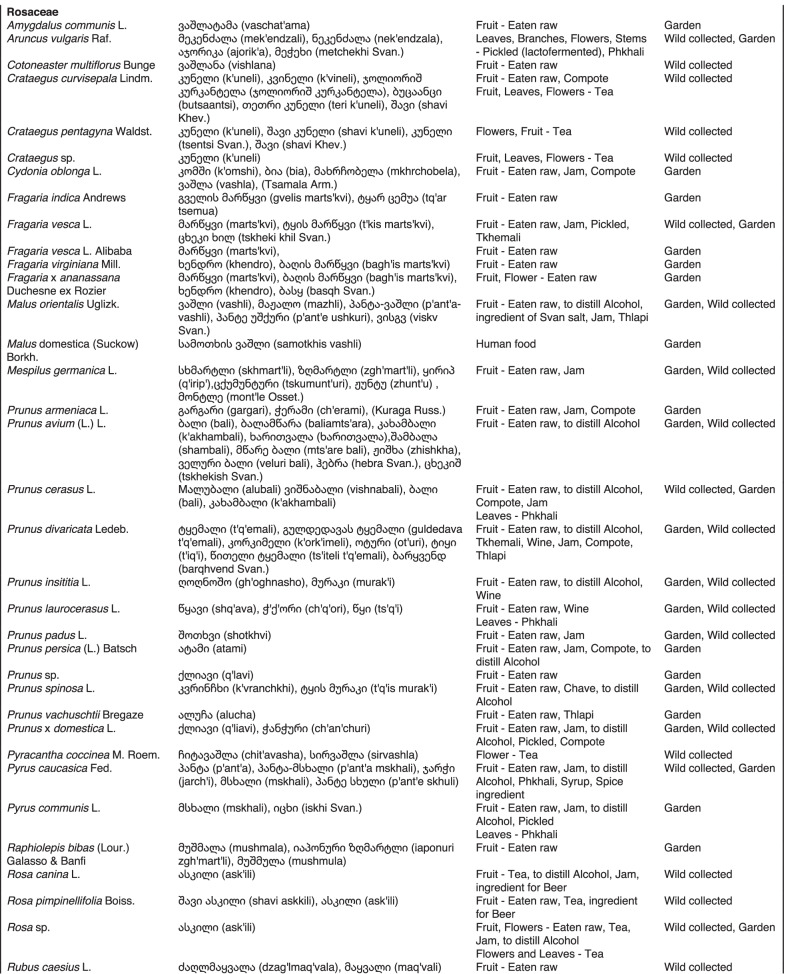

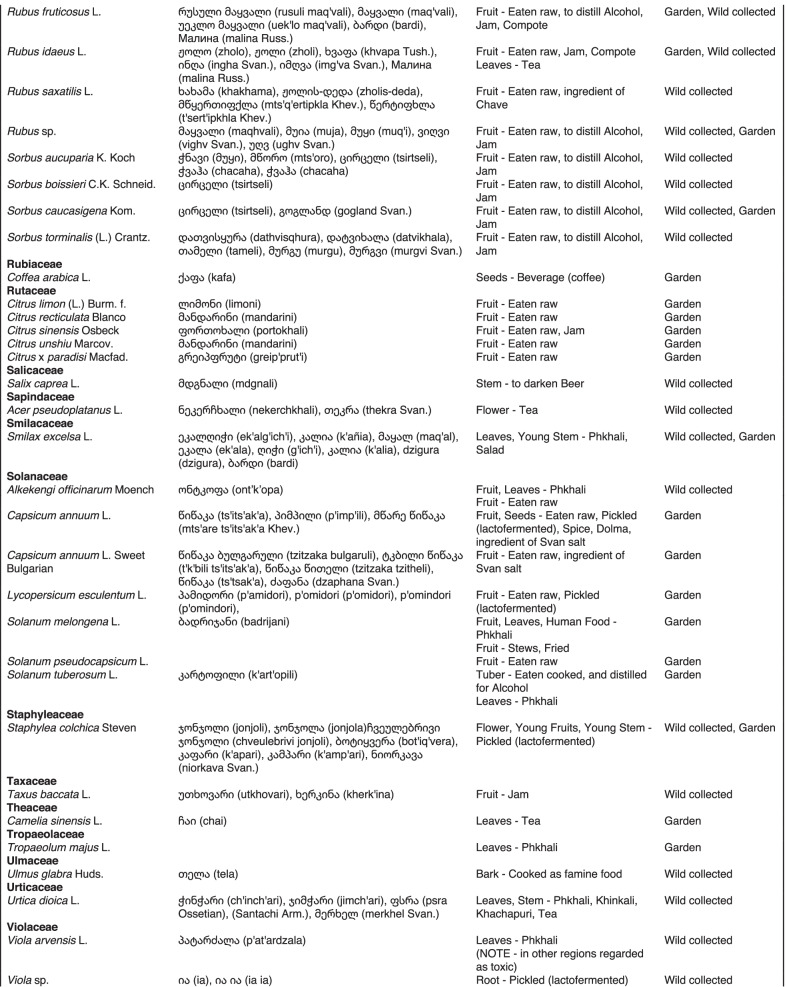

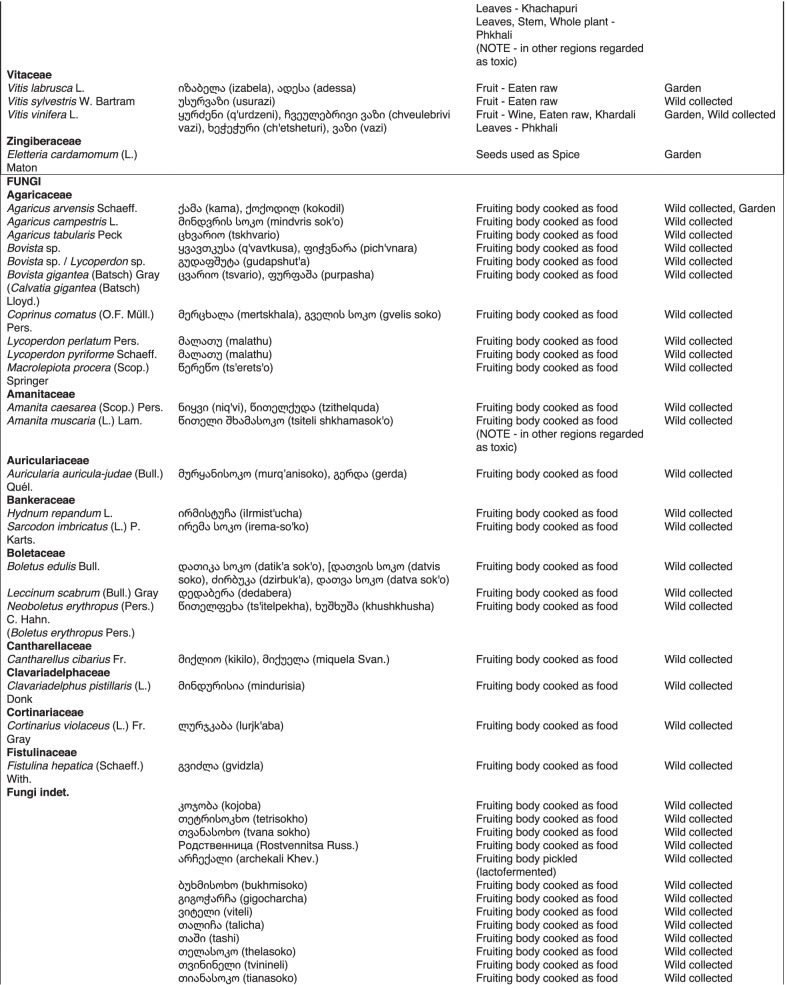

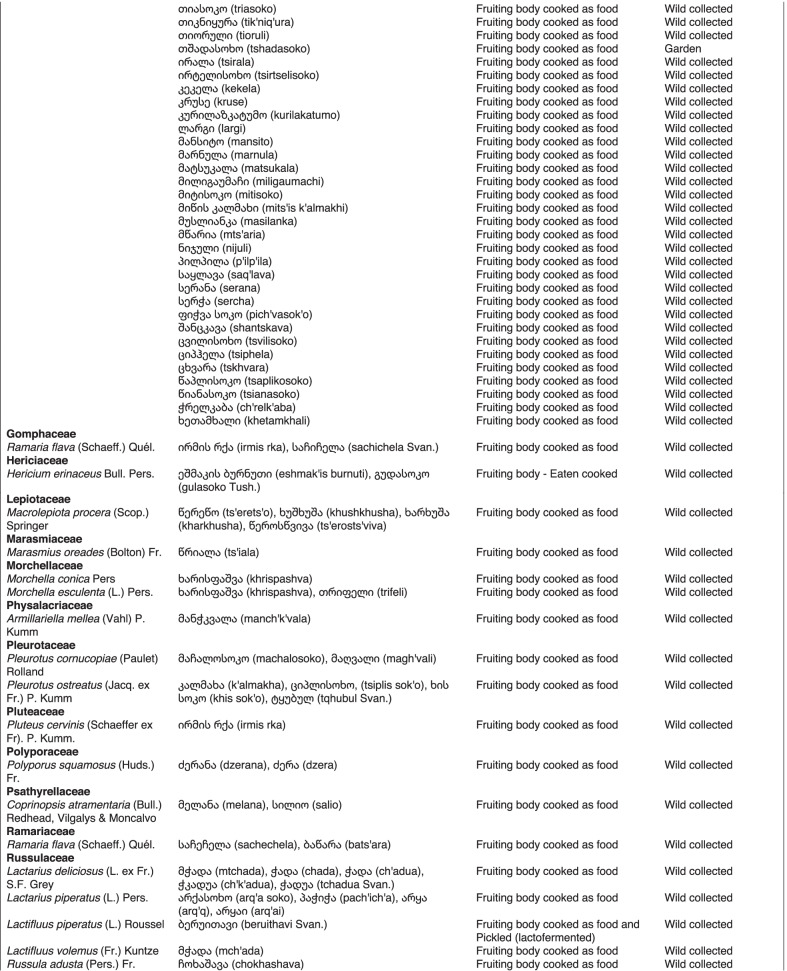

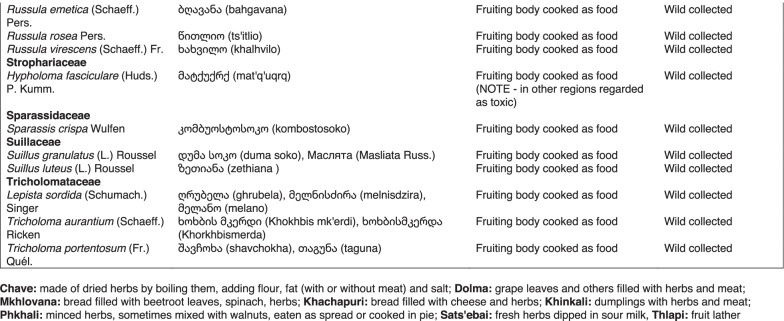
All Food plant and fungal species encountered in Georgia

### Data analysis

Data were tabulated using excel sheets and a combined matrix was constructed with plant entries in rows and plant data in columns including date, place, participant’s age and gender, interviewer, plant identity (Latin, Georgian vernacular, local names), the use category, which parts were used, and the source (garden or forest). We compared species diversity among groups of species (forest *versus* garden, various provinces) using sample-based rarefaction as well as widely used diversity indices: Dominance (*D*), Shannon (*H*), Evenness (*e^H/S*), Simpson index, (1 − *D*), Equitability (*J*), Fisher alpha, Berger–Parker (BP), given that no single index may sufficiently show the importance of certain species. Similarity of species composition among groups of plants were analyzed using non-metric multidimensional scaling (nMDS). All these analyses were performed using software PAST4.02 [71].

Test if the usage of plants based on family and genus, plant system used, and general and specific plant parts differ between regions and different altitudinal ranges. I predict that these components will be different, since there will be a different plant composition among regions and along an altitudinal gradient, and that different human communities have their own ethnobotany knowledge, even though they are from the same country.

We compared the usage of plants based on their (i) family and (ii) genus, (iii) system (root, shoot, or both), and (iv) general (vegetative, reproductive, or both) and (v) more specific (bark, branches, buds, bulb, cones, flowers, fruit, latex, leaves, resin, roots, seeds, shoots, silk, stem, timber, tuber, whole plant) parts used between regions and altitudinal ranges. We also compared (vi) for what purpose plants are used between regions and altitudinal ranges. We removed from our analyses any data that was not possible to make any further identification, such as plants identification above family, and uncertain plant parts. We also removed fungi from our analyses, and samples in which we had no more details about the purpose of usage of plants, i.e., in cases where plants were used as human food, but we did not know exactly for which kind of food. We considered regions and five altitudinal ranges (0–500 m, 501–1000 m, 1001–1500 m, 1501–2000 m, 2001–2500 m) as factors within our ordinations. We conducted non-metric multidimensional scaling (NMDS) followed by a permutational multivariate analysis of variance (PERMANOVA) with Euclidean distance and 999 permutations using the “RVAideMemoire” package [72].

## Results

**Fig. 2:**
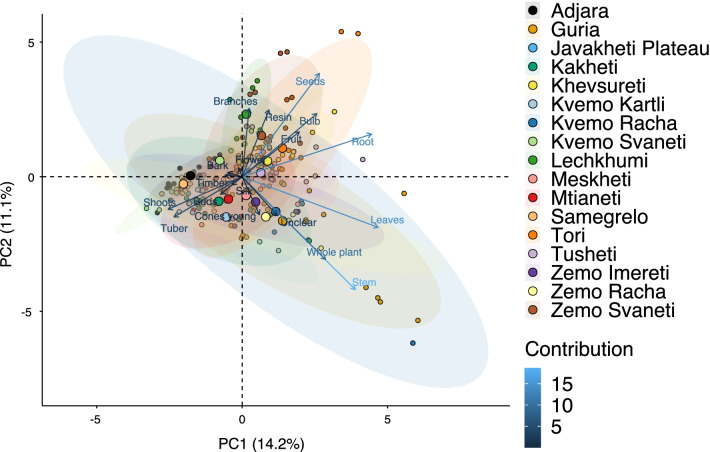
Principal component analysis comparing the usage of plants based on their specific parts (bark, branches, buds, bulb, cones, flowers, fruit, latex, leaves, resin, roots, seeds, shoots, silk, stem, timber, tuber, whole plant) used. Contribution represents how each family contributes to the overall dissimilarity between regions based on their distance on the ordination. Arrows represent the specific plant parts used, small dots the samples and larger dots the centroid of each region

The total number of taxa, mostly identified to species, was 527 (Tables 1 and 2, Appendix Tables 5, 6). Ninety-five species of fungi were consumed. Trees contributed 71 species (13.47%), Shrubs—43 (8.1%), Herbs—333 (60.32%), Climbers -5 (0.09%), and Fungi—95 (18.02%). Of all species 388 were wild, i.e., not cultivated, although some of them occurred on ruderal places and as weeds in gardens. In case of 20 vascular plants and 45 fungal species, the collected material did not allow a certain identification, and these species are thus indicated as "indet." in Table 1. Taxonomically, the difference between two food plant groups—garden versus wild ("forest")—was strongly pronounced even at family level. Only one plant species (*Piper nigrum* with four mentions) was bought in markets. Over 62% of the mentions (12,255) referred to cultivated plants, 7352 (37%) to wild collections, and some plants were found both collected in the wild and in gardens; however, this was a very small percentage (189 mentions, less than 1%). The great majority of mentions (> 99%) were either from families found either in gardens (62%) or in the wild (37%). Over 41% of all mentions referred to the use of fruits, 21% to leaves, about 7% to seeds, and 5% to fruiting bodies, leaves/stems and stems. Whole plants were only used very infrequently. Of all the families, Rosaceae, Apiaceae, Lamiaceae, Amaryllidaceae and Solanaceae showed the highest importance. At a generic level, *Allium*, *Pyrus*, *Malus* and *Brassica* received the highest number of use report. Only 30 species (6% of the total) represented 46% of all use mentions, but only *Malus orientalis* (3.5%), *Pyrus communis* (3.2%), and *Vitis vinifera* (2.7%) had over 2% of mentions, and *Chenopodium album* and *Urtica dioica* were the only not cultivated plants reaching over 1% of mentions. In most regions at all altitudinal ranges, the aboveground parts were mist frequently used (Fig. 2),

**Table 2:** Regions of our fieldwork and number of food plant mentions recorded

Region	Number of mentions
Guria	2125
Khevsureti	2012
Zemo Svaneti	1942
Adjara	1866
Tori	1750
Tusheti	1633
Kvemo Svaneti	1406
Kakheti	1085
Lechkhumi	1017
Samegrelo	853
Meskheti	776
Kvemo Racha	708
Javakheti	699
Kvemo Kartli	678
Zemo Imereti	631
Mtianeti	342
Zemo Racha	277

Most plants (65%) were eaten without complicated preparation, either raw (55%), or fried/cooked (e.g., 8% that were fungi). A full 5% of all mentioned plant-uses were for pickles / lactofermented (often stems), and a full 18% of all use reports were for *Phkhali* (boiled herb pie, especially in spring), 4% were used as spices, and around 2% for the distillation of alcohol. All other use categories (35) had much fewer mentions.

The richness of plant families was 66 in garden versus 97 families of wild plants, respectively, and the difference was highly significant. Other diversity indices also unequivocally pointed to a considerably more diverse family composition of wild versus garden plants as the differences between all the tested diversity indices appeared to be highly significant (Table 3).

**Table 3:** Plant family diversity assessed by various indices

Index	Garden	Wild	P-value
Dominance, *D*	0.096	0.053	0.0001
Shannon *H*	2.709	3.525	0.0001
Evenness e^*H/S*	0.227	0.346	0.0001
Simpson index, 1—*D*	0.904	0.947	0.0001
Equitability *J*	0.647	0.769	0.0001
Fisher *alpha*	9.168	15.9	0.0001
Berger–Parker, *BP*	0.219	0.166	0.0001

*P*-values are calculated using randomization tests (or Permutation test, software PAST 4.2)

**Fig. 3:**
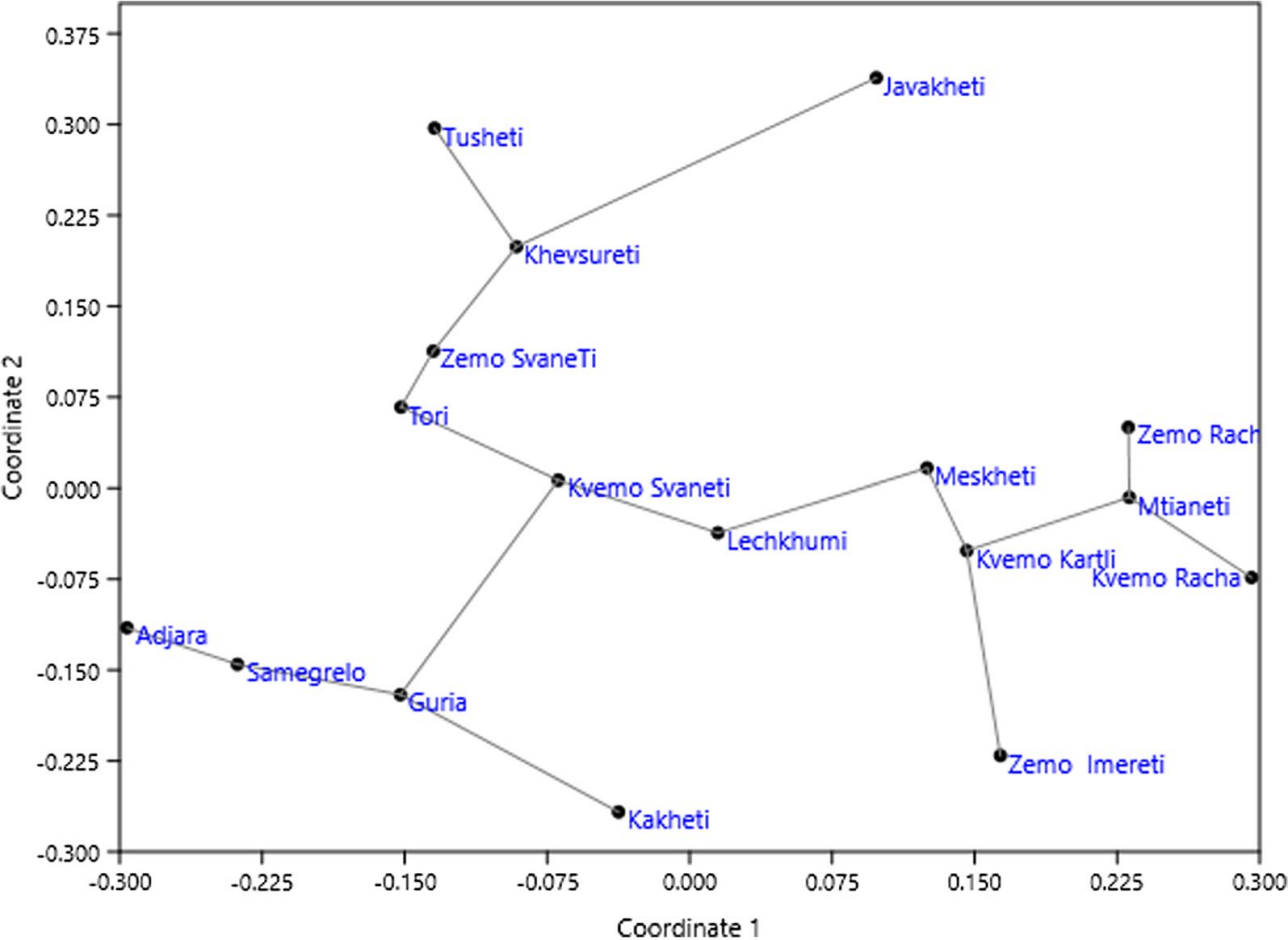
nMDS ordination of regions by garden food plant species composition

The regions of Georgia could be divided into three groups by the similarity of garden food plants as can be seen on the nMDS ordination graph (Fig. 3). This ordination seems to be influenced on the presence of large markets: Adjara, Samegrelo, Guria, and Kakheti which are lowland regions with large cities are joined by minimum distance versus Tori, Zemo Svaneti, Khevsureti, Tusheti and Javakheti, which are the most remote places. Kvemo Svaneti, Lechkhumi, Meskheti, Kvemo Kartli, Zemo Imereti, Zemo and Kvemo Racha, Mtianeti are moderately remote from large markets. The grouping of the regions closer to large markets might however have another distinct reason: Adjara, Samegrelo, Guria, and Kakheti are also the climatically warmest regions in Georgia, with the longest growing seasons. This allows the production of food plants almost all year round, and greatly reduces the dependency on foraging wild species.

For comparison, we assessed the usage of plants between regions based on their family, genus, specific parts used (root, shoot, or both) used, reproductive stages used (vegetative, reproductive, or both) and their specific parts used (bark, branches, buds, bulb, cones, flowers, fruit, latex, leaves, resin, roots, seeds, shoots, silk, stem, timber, tuber, whole plant), but at regional level and within different altitudinal ranges through non-multidimensional scaling (NMDS) followed by permutational multivariate analysis of variance (PERMANOVA) with 999 permutations and Euclidian distance. The detailed results are given in Table 4 and Appendix Tables 7, 8, 9, 10 and 11.

**Table 4:** Pairwise comparisons with FDR p-value adjustment method of the different variables evaluated (plant family, plant genus, system used, general plant parts used, specific plant parts used, the usage) between altitudinal ranges after significant PERMANOVA analysis (Table Permanova)

Plant family				
	0–500	1001–1500	1501–2000	2001–2500
1001–1500	0.0013			
1501–2000	0.0013	0.0013		
2001–2500	0.0013	0.0013	0.0013	
501–1000	0.0490	0.0044	0.0013	0.0013
*Plant genus*				
	0–500	1001–1500	1501–2000	2001–2500
1001–1500	0.0011			
1501–2000	0.0011	0.0011		
2001–2500	0.0011	0.0011	0.0011	
501–1000	0.0180	0.0011	0.0011	0.0011
*General plant parts used*				
	0–500	1001–1500	1501–2000	2001–2500
1001–1500	0.0300			
1501–2000	0.3550	0.0300		
2001–2500	0.4144	0.0300	0.3550	
501–1000	0.0420	0.6270	0.0833	0.0300
*General plant parts used*				
	0–500	1001–1500	1501–2000	2001–2500
1001–1500	0.0017			
1501–2000	0.0722	0.0017		
2001–2500	0.0017	0.0017	0.0017	
501–1000	0.0271	0.6840	0.0288	0.0017
*Specific plant parts used*				
	0–500	1001–1500	1501–2000	2001–2500
1001–1500	0.0017			
1501–2000	0.0025	0.0017		
2001–2500	0.0017	0.0017	0.0017	
501–1000	0.0222	0.6670	0.0025	0.0017
*Usage*				
	0–500	1001–1500	1501–2000	2001–2500
1001–1500	0.0133			
1501–2000	0.0050	0.0957		
2001–2500	0.0050	0.0840	0.3020	
501–1000	0.0450	0.2833	0.0917	0.1750

Analyses were based on Euclidean distance and 999 permutations

**Fig. 4:**
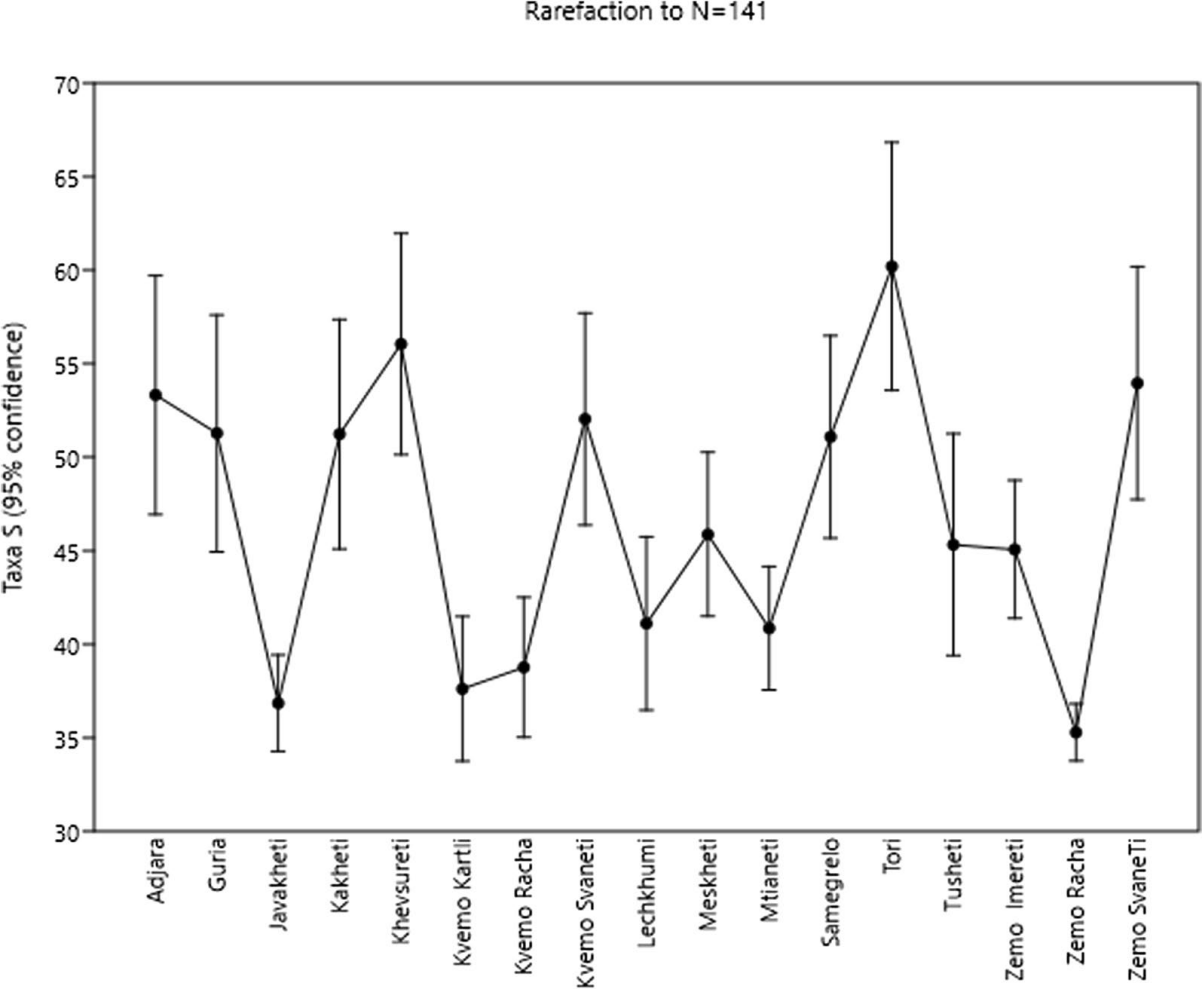
Rarefaction of species richness of the garden food plants across the regions

The regions varied strongly in their species richness, based on species used (Fig. 4). These differences also might reflect the remoteness from large markets and severity of local climate.

**Fig. 5:**
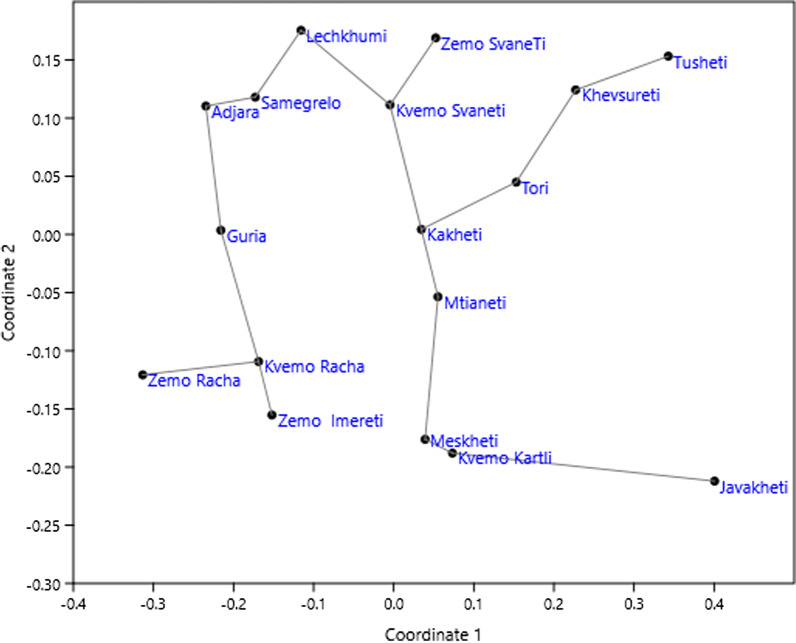
nMDS ordination of regions by wild food plant species composition

**Fig. 6:**
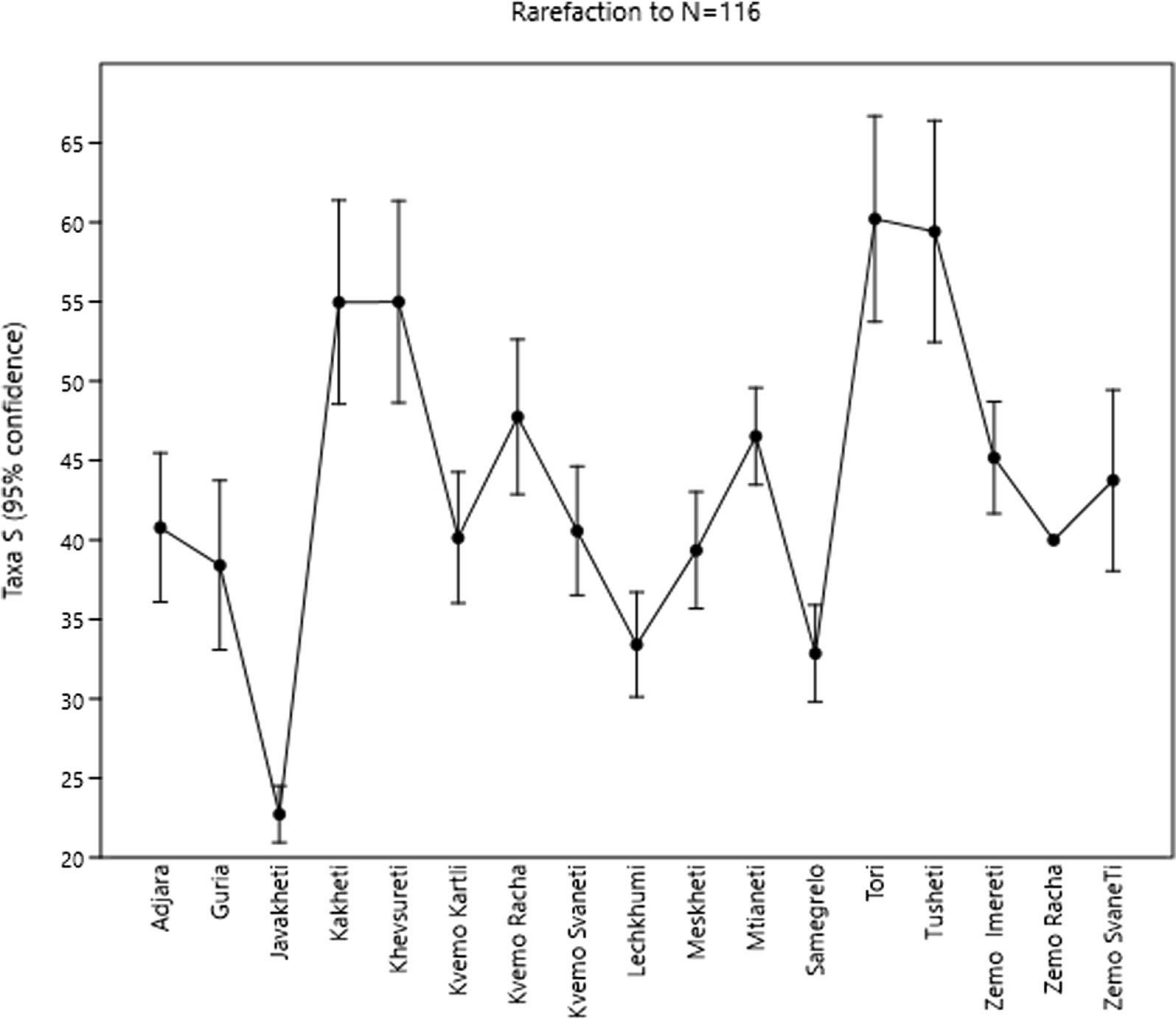
Rarefaction of species richness of the wild food plants across the regions

Relationships among the regions in the case of wild food plants show a different and clearer pattern (Fig. 5). Adjacent regions in particular cluster together (Kvemo Zemo Racha, and Zemo Imereti; Samegrelo, Guria, Adjara, Lechkhumi and Kvemo and Zemo Svaneti; Meskheti, Javakheti, Kvemo Kartli; Mtianeti, Kakheti, Khevsureti, Tusheti). Like in the case of the garden food plants, species diversity of the wild food plants mentioned varied strongly (Fig. 6). Climate and the need for of the use of wild food plants (especially in high altitude villages) play a role in this variation. As we already showed in various previous publications, language, cultural group, ethnicity, education, or gender of the participants had no impact on the main use of food plants, nor any other uses [41–50].

### Pkhali and Pickles—emblematic foods of the Caucasus

Of all food preparations the use of plants as ingredient of boiled herb preparations (mostly as  —gazapkhuli pkhali = Spring Pkhkali, as the first vitamin source after winter), and as lacto-fermented or vinegar-based pickles are probably the most emblematic ones in the Caucasus, given that almost 50% of all food mentions were for phkhali, and almost 12% for pickled plants, and 8% for teas.

**Fig. 7:**
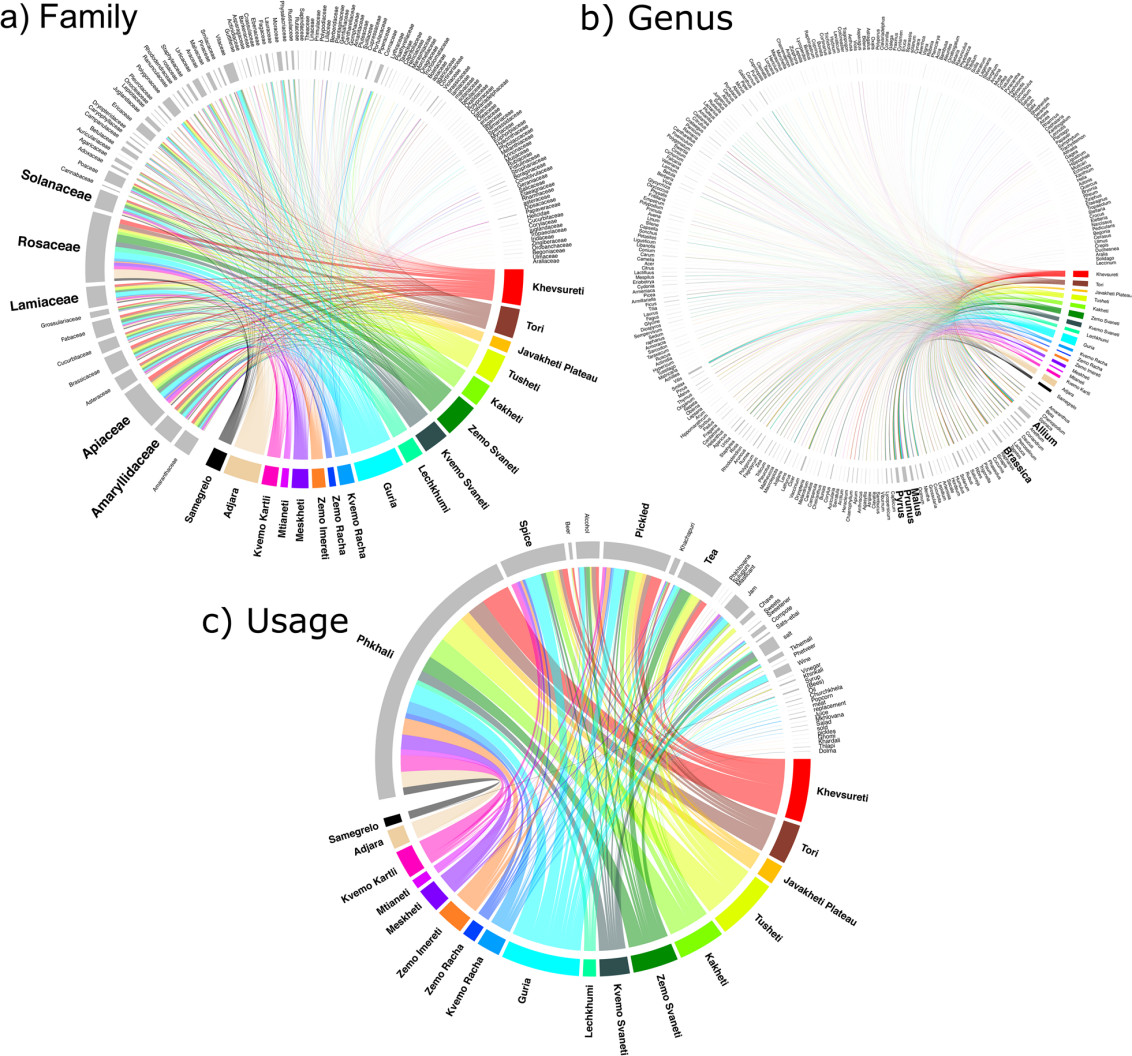
Relationship between families, genera and usage within regions

**Fig. 8:**
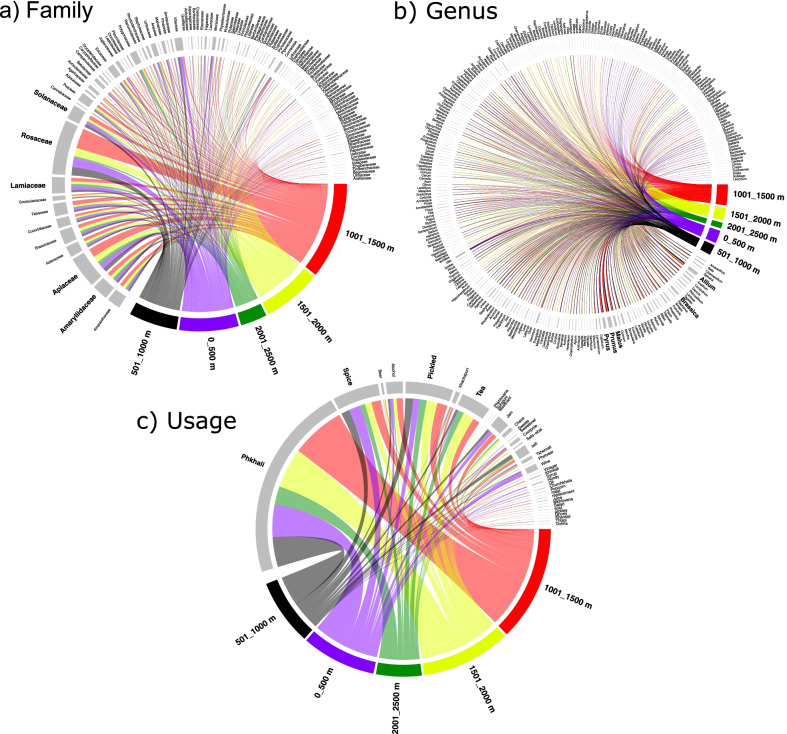
Relationship between families, genera and usage within the altitudinal gradients

**Fig. 9:**
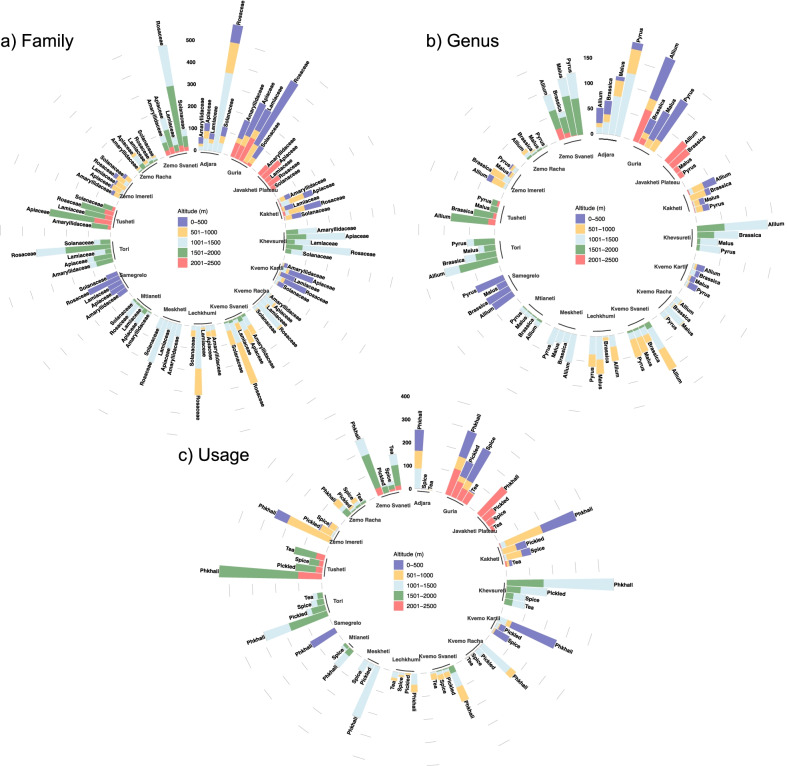
Absolute mention of families and genera by region and altitudinal distribution

While the overall distribution of families, genera and their uses were similar between regions, overall most species were used in Guria. However, the knowledge distribution was most uneven for these food categories (Fig. 7). The altitudinal range between 1001 and 1500 m, followed by 1501–2000 m were clearly predominant when it came to diversity of plants used as well as uses (Fig. 8). This very unequal distribution of the most important families/genera, as well as their respective uses is reflected in Fig. 9. The altitudinal differences do not necessarily indicate however that the respective species did not grow also at lower altitudes. They simply indicate that at lower altitudes the participants rather preferred other food plants, and due to a lack of necessity were not interested in wild harvesting greens.

Only 60% of participants reported making pickles / lactofermented preparations. Of these, over 16% each came from Zemo Imereti and Khevsureti, and 12% each from Zvemo Svaneti, the Javakheti-Plateau, and Guria. The first regions represent all high altitude—short growing season areas, where the population does need to preserve food for winter. Guria is relatively warm—but very wet and snow-rich, which also might explain the prevalence of pickles. No participants whatsoever from Adjara, Samegrelo (the most subtropical regions) and Mtianeti (close to the capital Tbilisi) reported making pickles. Unsurprising, Kakhetians were also not enthusiastic about this form of preparation, because Kahketi is also a region famous for its large agricultural production. In contrast, in Tori and Tusheti there are simply less products that can be pickled. Preferred species (of a total of 79) for pickles were mostly Amaranthaceae (*Amaranthus, Chenopdium*), Apiaceae (especially the stems of *Anthriscus*, *Chaerophyllum* and *Heracleum* were pickled, but also, stems of *Conium maculatum*), Amaryllidaceae (all *Allium* species), and Polygonaceae (*Polygonum* and *Rumex*). In addition, *Aruncus vulgaris* (Rosaceae), *Stapyllea colchica* (Staphyleaceae). All of these were more important as pickles than "traditional European style species (*Cucumis sativus*, *Capsicum* etc.). The fermentation of the ferns *Mattheucia struthiopteris* (Onocleaceae) and *Dryopteris filix-mas* (Dryopteridaceae) was similar to what we observed, e.g., in the Himalayas.

The participants clearly indicated that some plants (e.g., *Conium maculatum*, *Dryopteris filix-mas*, *Galanthus* sp., *Narcissus* sp.) needed careful preparations, due to possible toxicity. However, given that these species might have even higher toxicity in other regions, e.g., Central Europe, the authors decided to not elaborate any further on preparation methods, given that these might not be sufficient to remediate toxicity of the same species outside the Caucasus.

In case of Pkhali, over 93% of all participants—from all regions—reported to use such boiled herbs, normally in Spring. This was surprising, as we had expected much more limited use in the climatically favorable regions. Nevertheless, Zemo Imereti (19% of all Phkhali preparations), Tori and Kvemo Racha (16% each), Tusheti (15%) and Khevsureti (14%)—all mountain regions with long winters, stood out as the real "herb eater" areas. In contrast to the pickled species, essentially only young leaves were used for pkhali, with great emphasis on the same families indicated in pickles. (All pickled plant species were also used for phkhali.) The overall number of species fused or pkhali was however much higher (197). The elaboration of phkhali often involves many steps to reduce the toxicity of species used, and in most cases a wide variety of herbs are included in each preparation. Interesting examples for the use of toxic species included the leaves of *Solanum tuberosum*, *Veratrum lobelianum* and *Viola* sp. *Solanum tuberosum* leaves for example are regarded as toxic worldwide, but are being eaten in the Caucasus and Albania [48]. *Veratrum album* (closely related to *Veratrum lobelianum*, and growing especially in Europe, is highly toxic), and *Viola* sp. (although especially the flowers are widely used in gastronomy) contains toxic Saponins. In all cases careful preparation was mentioned to make these species palatable. The authors explicitly decided to not give any recipes, given that many of the species are widespread, and compound composition—and with it possible toxic effects—might vary across the distribution range, so that a preparation method that sufficiently reduces toxicity in the Caucasus might not necessary be applicable in other areas.

## Discussion

The use of food plant in Georgia while varied showed distinct overlap with other studies. However, the number of food plant species used—both cultivated and foraged in this rather small territory—was far higher than in most published studies from either wider region or the Mediterranean and Eurasia. Of all species, 388 were wild/wild collected, although a few of them also occurred as weeds in gardens. Even when deducting the fungal species (95), the remaining 293 vascular plant species are a mostly a much higher number than found in any other study in the wider region [73–106] (73:148 species; 74:87 species; 75:41 species; 76:40 species; 77:276 species; 78:119 species; 79:84 species; 80:68 species; 81:30–100 species for different European regions; 82:112 species; 83:139 species; 84:49 species; 85:15 species (although focusing on weeds only); 86:78 species; 87:419 species for all of Spain; 88:36; 89:77 species; 90:40 species; 91:11 species; 92:48 species; 93:83 species; 94:105 species; 95:73 species; 96:47 species; 97:115 species; 98:67 species; 99:78 species; 100:79 species; 101:35 species; 102:52 species; 103:63 species; 104:80 species; 105:88 species; 106:51 species).

Interestingly, even studies conducted in pastoralist cultures well-known for their use of wild foraged plants for food, e.g., in relatively close-by Kurdistan [107, 108] (107:54 species; 108:65 species), and Turkey [109] with 74 species showed a much more limited use of plants for food, even when not considering the 20% of taxa found in Georgia that were fungi. In many areas of the same cultural space, e.g., Dagestan [110] with 24 species, Azerbaijan [111, 112] (111:72 species; 112:73 species) and Amenia [113] with 66 species) the use of wild plants for food has been shown as in steep decline, although a strong signature of food plant use could still be found in markets of the Armenian capital Yerevan [114] with 148 species.

Outside the region, e.g., in China, it has been shown that typical agricultural communities use a very large number of wild species [115–117] (115: 185 vascular plant species and 17 fungal folk taxa; 116: 224 species; 117: 168 species). In many cases, however, wild plant use fell far short from the species numbers found in the Caucasus, e.g., [118–120] (118: 81 species; 119: 59 species; 120: 54 vascular plant species and 22 fungi).

The use of food species was not closely related to different vegetation zones in Georgia. This is a specific feature of food plants and differs from the use of plants in other categories, as has been previously shown [38–50].

The large number of species used in comparison with other areas confirmed our first hypothesis that given the long tradition of plant use, and the isolation under Soviet rule, plant use both based on home gardens and wild harvesting would be more pronounced in Georgia than in the wider region. In addition, the very large number of wild vegetables in Georgia might underline the hypothesis that the use of such wild "greens" is a byproduct of the Neolithic revolution, given that the region is indeed a cradle of agriculture as indicated previously [9, 13, 14].

We found a rather widespread use of foodplants across Georgia, which can partly be explained by mixture of populations from varied regions through migration and Soviet population moves, which also confirmed our hypothesis that food plant use knowledge would be widely and equally spread in most of Georgia.

Finally, we indeed found that in the very fertile agricultural regions in Eastern (Kakheti) and Western (lower Ajara, Samegrelo) Georgia, plant use knowledge was indeed more limited. However, this does not explicitly confirm our third hypothesis that such regions would show knowledge loss, as the limited use of plants may already have persisted a long time, and historic comparative data are not available.

## Conclusions

This study reported on 535 plant and fungal taxa used in Georgia as food. As many mountain regions all over the world, the rural areas of the Georgian Caucasus have suffered a constant population decline for decades, due to harsh economic conditions and lack of modern infrastructure [1, 24, 121–124]. While this has greatly accelerated the loss of traditional agricultural practices, it seems to have affected the use of wild gathered food plants as well as species grown in home gardens to a much more limited extent in Georgia. The home gardens in Georgia clearly continue serving as socio-ecological memory, and an irreplaceable part of Georgian culture, rather than the widely growing popularity of gardening and foraging found all over Europe [125]. The great variety of food plant species used in the Georgian Caucasus provides a reservoir for food security for the region, as well as a source of important food plant germplasm for international agriculture. This greatly underlines the importance of Georgia as an ancient center of crop domestication and diversification, making Georgia clearly one of the most diverse food plant cultures in wider Eurasia, and the center of what we would like to coin as "Caucasus—Asia Minor—Balkans cultural complex."

## Data Availability

The anonymized raw data are deposited under Open Science Network: https://osf.io/9kdtw/?view_only=93a8748c003f4770bc4a2bb332647429

## Appendix

See Tables 5, 6, 7, 8, 9, 10 and 11.

**Table 5:** Species of identified food plants and fungi and the number of their mentions recorded

Plant / Fungal family	Plant / Fungal species	Mentions
Actinidiaceae	*Actinidia callosa* Lindl	28
Adoxaceae	*Sambucus ebulus* L	83
Adoxaceae	*Sambucus nigra* L	9
Adoxaceae	*Viburnum lantana* L	21
Adoxaceae	*Viburnum opulus* L	21
Agaricaceae	*Agaricus arvensis* Schaeff	165
Agaricaceae	*Agaricus campestris* L	4
Agaricaceae	*Agaricus tabularis* Peck	1
Agaricaceae	*Bovista* sp.	12
Agaricaceae	*Bovista* sp. / *Lycoperdon* sp.	4
Agaricaceae	*Clavatia gigantea* (Batsch) Rostk	14
Agaricaceae	*Coprinus comatus* (O.F. Müll.) Pers	2
Agaricaceae	*Lycoperdon perlatum* Pers. / *Lycoperdon pyriforme* Schaeff	2
Amanitaceae	*Amanita caesarea* (Scop.) Pers	15
Amanitaceae	*Amanita muscaria* (L.) Lam	1
Amaranthaceae	*Amaranthus palmeri* S. Watson	16
Amaranthaceae	Amaranthus paniculatus L	24
Amaranthaceae	*Amaranthus retroflexus* L	132
Amaranthaceae	*Amaranthus speciosus* L	1
Amaranthaceae	*Amaranthus spinosus* L	3
Amaranthaceae	*Atriplex hortensis* L	35
Amaranthaceae	*Beta vulgaris* L	311
Amaranthaceae	*Beta vulgaris* L. ssp. *cicla* (L.) Moq	36
Amaranthaceae	Beta *vulgaris* L. ssp. *esculenta* (Salisb.) Gürke var. *altissima* Rössig. = Beta *vulgaris saccharifera* Alef	3
Amaranthaceae	*Chenopodium album* L	203
Amaranthaceae	*Chenopodium bonus-henricus* L	1
Amaranthaceae	*Chenopodium foliosum* (Moench) Asch	35
Amaranthaceae	*Chenopodium* sp.	1
Amaranthaceae	*Spinacia oleracea* L	44
Amaryllidaceae	*Allium ampeloprasum* L	3
Amaryllidaceae	*Allium ascalonicum* L	7
Amaryllidaceae	*Allium atroviolaceum* Boiss	10
Amaryllidaceae	*Allium cepa* L	309
Amaryllidaceae	*Allium fistulosum* L	97
Amaryllidaceae	*Allium kunthianum* Vved	2
Amaryllidaceae	*Allium ponticum* Miscz	5
Amaryllidaceae	*Allium porrum* L	56
Amaryllidaceae	*Allium rotundum* L	20
Amaryllidaceae	*Allium sativu*m L	340
Amaryllidaceae	*Allium* sp.	3
Amaryllidaceae	*Allium ursinum* L	54
Amaryllidaceae	*Allium victorialis* L	231
Amaryllidaceae	*Galanthus* sp.	10
Amaryllidaceae	*Galanthus woronowii* Losinsk	3
Amaryllidaceae	*Narcissus* sp.	5
Annonaceae	*Annona cherimola* Mill	1
Apiaceae	*Aethusa cynapium* L	1
Apiaceae	*Agasyllis latifolia* (Bieb.) Boiss	91
Apiaceae	*Anethum graveolens* L	301
Apiaceae	*Angelica tatianae* Bordz	2
Apiaceae	*Anthriscus cerefolium* (L.) Hoffm	4
Apiaceae	*Anthriscus nemorosus* (M. Bieb.) Spreng	16
Apiaceae	*Anthriscus sylvestris* L	15
Apiaceae	*Apium graveolens* L	128
Apiaceae	*Carum carvi* L	60
Apiaceae	*Chaerophyllum aureum* L	16
Apiaceae	*Chaerophyllum bulbosum* L	10
Apiaceae	*Chaerophyllum caucasicum* (Fisch.) B. Schischk	95
Apiaceae	*Conium maculatum* L	10
Apiaceae	*Coriandrum sativum* L	348
Apiaceae	*Daucus carota* L. ssp. *sativus*	251
Apiaceae	*Falcaria sioides* Asch	1
Apiaceae	*Falcaria vulgaris* Bernh	25
Apiaceae	*Foeniculum vulgare* Mill	79
Apiaceae	*Heracleum asperum* M. Bieb	30
Apiaceae	*Heracleum leskovii* Grossh	5
Apiaceae	*Heracleum* sect. *villosum*	2
Apiaceae	*Heracleum sosnowskyi* Manden	59
Apiaceae	*Heracleum* sp.	36
Apiaceae	*Heracleum wilhelmsii* Fisch. & Ave-Lall	30
Apiaceae	*Hippomarathrum crispum* (Pers.) Boiss	4
Apiaceae	*Hippomarathrum microcarpum* Petrov	1
Apiaceae	*Levisticum officinale* W.D.J. Koch	2
Apiaceae	*Libanotis transcaucasica* Schischk	15
Apiaceae	*Ligusticum alatum* Spreng	4
Apiaceae	*Petroselinum crispum* (Mill.) Fuss	268
Apiaceae	*Xanthogalum purpurascens* Avé-Lall	3
Araceae	*Arum albispatum* Stev. ex Ledeb	2
Araceae	*Arum orientale* M. Bieb	7
Araceae	*Arum* sp.	20
Araliaceae	*Aralia spinosa* L	1
Asparagaceae	*Asparagus officinalis* L	30
Asparagaceae	*Asparagus* sp.	4
Asparagaceae	*Muscari sosnowskyi* Schchian	2
Asparagaceae	*Ornithogalum woronowii* Kasch	2
Asparagaceae	*Polygonatum glaberrimum* C. Koch	13
Asparagaceae	*Ruscus colchicus* Yeo	1
Asparagaceae	*Ruscus hypophyllum* L	2
Asparagaceae	*Scilla siberica* Andrews	6
Asparagaceae	*Scilla* sp.	6
Asteraceae	*Achillea grandiflora* M. Bieb	1
Asteraceae	*Achillea millefolium* L	5
Asteraceae	*Arctium lappa* L	32
Asteraceae	*Artemisia absinthium* L	8
Asteraceae	*Artemisia dracunculus* L	125
Asteraceae	*Artemisia vulgaris* L	3
Asteraceae	*Bidens tripartida* L	4
Asteraceae	*Cichorium intybus* L	11
Asteraceae	*Cirsium incanum* (S.G. Gmel.) Fisch. ex M. Bieb	13
Asteraceae	*Cirsium* sp.	5
Asteraceae	*Cirsium vulgare* L	3
Asteraceae	*Crepis* sp.	3
Asteraceae	*Cynara cardunculus* L	6
Asteraceae	*Echinops* sp.	2
Asteraceae	*Eruca sativa* Mill	12
Asteraceae	*Helianthus annuus* L	17
Asteraceae	*Helianthus tuberosus* L	17
Asteraceae	*Lactuca sativa* L	165
Asteraceae	*Lactuca sativa* L. "greek"	1
Asteraceae	*Lactuca serriola* L	17
Asteraceae	*Lapsana communis* L	9
Asteraceae	*Lapsana grandiflora* M. Bieb	2
Asteraceae	*Matricaria chamomilla* L	5
Asteraceae	*Petasites albus* (L.) Gaertn	14
Asteraceae	*Petasites hybridus* (L.) G. Gaert, B. Mey. & Scherb	51
Asteraceae	*Serratula quinquefolia* Bieb. ex Willd	20
Asteraceae	*Solidago canadensis* L	4
Asteraceae	*Sonchus asper* (L.) Hill	7
Asteraceae	*Stevia* sp.	2
Asteraceae	*Tagetes patula* L	114
Asteraceae	*Taraxacum confusum* Schischk	2
Asteraceae	*Taraxacum officinale* Wigg	41
Asteraceae	*Tragopogon* sp.	19
Asteraceae	*Tussilago farfara* L	1
Asteraceae	*Xanthium strumarium* L	3
Auriculariaceae	*Auricularia auricula-judae* (Bull.) Quél	10
Bankeraceae	*Hydnum repandum* Fr	2
Bankeraceae	*Sarcodon imbricatus* (L.) P. Karts	8
Begoniaceae	*Begonia rex* Putz	10
Berberidaceae	*Berberis vulgaris* L	54
Betulaceae	*Alnus barbata* C.A. Mey	1
Betulaceae	*Betula litwinowii* Doluch	3
Betulaceae	*Betula* sp.	2
Betulaceae	*Corylus avellana* L. / *C. pontica* K. Koch	200
Betulaceae	*Corylus iberica* L	4
Boletaceae	*Boletus edulis* Bull	16
Boletaceae	*Neoboletus erythropus* (Pers.) C. Hahn	2
Boletaceae	*Leccinum scabrum* (Bull.) Gray	3
Boraginaceae	*Myosotis* sp.	2
Boraginaceae	*Symphytum grandiflorum* DC	14
Boraginaceae	*Trachystemon orientalis* (L.) G. Don	6
Brassicaceae	*Armoracia rusticana* (G. Gaertn.) B. Mey. & Scherb	33
Brassicaceae	*Brassica campestris* L	1
Brassicaceae	*Brassica campestris* L. ssp. *oleifera* DC	9
Brassicaceae	*Brassica juncea* (L.) Czern	3
Brassicaceae	*Brassica montana* Pourr	36
Brassicaceae	*Brassica oleracea* L	361
Brassicaceae	*Brassica oleracea* L. red	9
Brassicaceae	*Brassica oleracea* L. var. *botrytis* cauliflower	25
Brassicaceae	*Brassica oleracea* L. var. *gemmifera* Brussles Sprouts	1
Brassicaceae	*Brassica oleracea* L. var. *gongylodes*	47
Brassicaceae	*Brassica oleracea* L. var. *italica*	21
Brassicaceae	*Brassica rapa* L. subsp. *rapifera* Metzger	67
Brassicaceae	*Brassica rapa* var. *rapa* L	45
Brassicaceae	*Bunias orientalis* L	27
Brassicaceae	*Capsella bursa-pastoris* L	26
Brassicaceae	*Cardamine hirsuta* L	10
Brassicaceae	*Cheiranthus cheiri* L	1
Brassicaceae	*Lepidium sativum* L	52
Brassicaceae	*Raphanus raphanistrum* subsp. *sativus* (L.) Domin	17
Brassicaceae	*Raphanus sativus* L. var. *major*	179
Brassicaceae	*Raphinastrum rugosum* L. All	13
Brassicaceae	*Sinapis arvensis* L	15
Campanulaceae	*Campanula alliariifolia* Wild	2
Campanulaceae	*Campanula biebersteiniana* Roem. & Schult	1
Campanulaceae	*Campanula glomerata* L	7
Campanulaceae	*Campanula lactiflora* M. Bieb	70
Campanulaceae	*Campanula latifolia* L	11
Campanulaceae	*Campanula rapunculoides* L	20
Cannabaceae	*Cannabis sativa* L	30
Cannabaceae	*Humulus lupulus* L	22
Cantharellaceae	*Cantharellus cibarius* Fr	36
Caprifoliaceae	*Lonicera caucasica* Pall	3
Caryophyllaceae	*Melandrium balansae* Boiss	5
Caryophyllaceae	*Melandrium boissieri* Schischk	9
Caryophyllaceae	*Melandrium* sp.	5
Caryophyllaceae	*Oberna wallichiana* (Klotzsch) Ikonn	3
Caryophyllaceae	*Silene lacera* Steven	15
Caryophyllaceae	*Silene sibirica* (L.) Pers	2
Caryophyllaceae	*Silene wallachiana* Klotzsch	9
Caryophyllaceae	*Stellaria media* (L.) Vill	9
Clavariadelphaceae	*Clavariadelphus pistillaris* (L.) Donk	5
Convolvulaceae	*Convolvulus arvensis* L	17
Cornaceae	*Swida australis* (C.A. Mey.) Pojark ex Grossh	5
Cortinariaceae	*Cortinarius violaceus* (L.) Fr. Gray	1
Crassulaceae	*Sedum caucasicum* Boriss	8
Crassulaceae	*Sedum oppositifolium* Sims	5
Crassulaceae	*Sedum stoloniferum* Gmel	5
Crassulaceae	*Sempervivum caucasicum* Rupr. ex Boiss	14
Cucurbitaceae	*Bryonia dioica* Jacq	3
Cucurbitaceae	*Citrullus lanatus* (Thunb.) Matsum. & Nakai	16
Cucurbitaceae	*Cucumis melo* L	4
Cucurbitaceae	*Cucumis sativus* L	363
Cucurbitaceae	*Cucurbita maxima* L	14
Cucurbitaceae	*Cucurbita pepo* L	201
Cucurbitaceae	*Cucurbita pepo* L. var. *giromontia*	39
Cucurbitaceae	*Cucurbita pepo* L. var. *patisson*	9
Cucurbitaceae	*Cucurbita* sp.	14
Cucurbitaceae	*Lagenaria siceraria* (Molina) Standl	2
Cupressaceae	*Junperus sabina* L	2
Dipsacaceae	*Cephalaria gigantea* (Ledeb.) Bobrov	1
Dryopteridaceae	*Dryopteris filix-mas* (L.) Schott	35
Ebenaceae	*Diospyros lotus* L	54
Ebenaceae	*Diospyros* sp.	4
Ebenaceae	*Diospyros virginiana* L	5
Elaeagnaceae	*Elaeagnus* sp.	3
Elaeagnaceae	*Hippophaë rhamnoides* L	3
Elaeagnaceae	*Shepherdia argentea* Nutt	1
Elaeagnaceae	*Shepherdia* sp.	3
Ericaceae	*Empetrum hermaphroditum* Hagerup	21
Ericaceae	*Oxycoccus quadripetalus* Gilib	1
Ericaceae	*Vaccinium arctostaphylos* L	190
Ericaceae	*Vaccinium myrtillus* L	209
Ericaceae	Vaccinium sp.	4
Ericaceae	*Vaccinium uliginosum* L	2
Ericaceae	*Vaccinium vitis*-idaea L	49
Euphorbiaceae	*Aleurites moluccanua* (L.) Willd	1
Fabaceae	*Astragalus caucasisus* Pall	1
fabaceae	*Cicer arietinum* L	25
Fabaceae	*Coronilla varia* L	5
Fabaceae	*Galega orientalis* Lam	9
Fabaceae	*Glycine max* (L.) Merr	35
Fabaceae	*Glycyrrhiza glabra* L	1
Fabaceae	*Lathyrus roseus* Steven	42
Fabaceae	*Lathyrus tuberosus* L	3
Fabaceae	*Lens cornicularis* L	16
Fabaceae	*Phaseolus sativus* L	270
Fabaceae	*Phaseolus vulgaris* L	86
Fabaceae	*Pisum sativum* L	66
Fabaceae	*Robinia pseudoacacia* L	45
Fabaceae	*Trifolium* sp.	5
Fabaceae	*Trigonella caerulea* (L.) Ser	173
Fabaceae	*Vicia faba* L	54
Fabaceae	*Vicia sativa* L	1
Fabaceae	*Vigna angularis* (Willd.) Ohwi & H. Ohashi	1
Fagaceae	*Castanea sativa* Mill	79
Fagaceae	*Fagus orientalis* Lipsky	53
Fagaceae	*Quercus iberica* M. Bieb	9
Fistulinaceae	*Fistulina hepatica* (Schaeff.) With	6
Fungi	Unidentified fungus	227
Gentianaceae	*Swertia iberica* Fisch & C.A. Mey	1
Geraniaceae	*Erodium cicutarium* (L.) L'Hér. ex Aiton	4
Geraniaceae	*Geranium robertianum* L	3
Geraniaceae	*Geranium* sp.	6
Grossulariaceae	*Grossularia reclinata* (L.) Mill	27
Grossulariaceae	*Ribes biebersteinii* Berl. ex DC	59
Grossulariaceae	*Ribes grossularia* L	22
Grossulariaceae	*Ribes nigrum* L	73
Grossulariaceae	*Ribes orientale* Desf	4
Grossulariaceae	*Ribes rubrum* L	103
Grossulariaceae	*Ribes* sp.	24
Grossulariaceae	*Ribes uva-crispa* L	13
Guttiferae	*Hypericum perforatum* L	22
Hericiaceae	*Hericium erinaceus* (Bull.) Pers	1
Iridaceae	*Crocus sativus* L	9
Juglandaceae	*Juglans mandshurica* Maxim	7
Juglandaceae	*Juglans regia* L	235
Juglandaceae	*Pterocarya pterocarpa* (Michx.) Kunth ex Iljinsk	7
Lamiaceae	*Lamium album* L	32
Lamiaceae	*Lamium purpureum* L	6
Lamiaceae	*Leonotis leonurus* (L.) R. Br	1
Lamiaceae	*Mentha aquatica* L	3
Lamiaceae	*Mentha longifolia* (L.) L	158
Lamiaceae	*Mentha pulegium* L	81
Lamiaceae	*Mentha* sp.	8
Lamiaceae	*Mentha* x *piperita* L	143
Lamiaceae	*Nepeta mussinii* Spreng	2
Lamiaceae	*Ocimum basilicum* L	198
Lamiaceae	*Ocimum basilicum* var. *purpurascens* Benth	8
Lamiaceae	*Origanum vulgare* L	50
Lamiaceae	*Salvia verticillata* L	3
Lamiaceae	*Satureja hortensis* L	92
Lamiaceae	*Satureja laxiflora* K. Koch	7
Lamiaceae	*Satureja spicigera* Boiss	31
Lamiaceae	*Thymus caucasicus* Willd. ex Benth	30
Lamiaceae	*Thymus colinus* Bieb	21
Lamiaceae	*Thymus* sp.	29
lamiaceae	*Thymus transcaucasicus* Ronninger	17
Lamiaceae	*Ziziphora pushkinii* Adams	18
Lamiaceae	*Ziziphora serpyllacea* M. Bieb	16
Lauraceae	*Laurus nobilis* L	25
Lauraceae	*Persea americana* Mill	2
Lepiotaceae	*Macrolepiota procera* (Scop.) Springer	51
Liliaceae	*Fritillaria lutea* Mill	11
Liliaceae	*Gagea* sp.	3
Liliaceae	*Lilium* sp.	1
Liliaceae	*Lilium szovitsianum* Fisch. & Avé-Lall	11
Liliaceae	*Ornithogalum woronowii* Kasch	6
Linaceae	*Linum usitatissimum* L	7
Lythraceae	*Punica granatum* L	32
Malvaceae	*Alcea rosea* L	1
Malvaceae	*Althaea* spp.	11
Malvaceae	*Malva neglecta* L	38
Malvaceae	*Malva sylvestris* L	10
Malvaceae	*Malva sylvestris* L. / *M. neglecta* L	59
Malvaceae	*Tilia begonifolia* Stev	2
Malvaceae	*Tilia caucasica* Rupr	49
Marasmiaceae	*Marasmius oreades* (Bolton) Fr	12
Melanthiaceae	*Veratrum lobelianum* Bernh	5
Moraceae	*Ficus carica* L	142
Moraceae	*Morus alba* L	99
Moraceae	*Morus nigra* L	7
Morchellaceae	*Morchella conica* Pers	1
Morchellaceae	*Morchella esculenta* (L.) Pers	12
Musaceae	*Musa* x *paradisiaca* L	3
Myrtaceae	*Acca sellowiana* (O. Berg.) Burret	11
Oleaceae	*Fraxinus excelsior* L	5
Oleaceae	*Ligustrum vulgare* L	2
Onagraceae	*Chamenaerion angustifolium* (L.) Holub	1
Onocleaceae	*Mattheuccia struthiopteris* (L.) Todd	35
Orobanchaceae	*Pedicularis* sp.	5
Oxalidaceae	*Averrhoa carambola* L	1
Oxalidaceae	*Oxalis acetosela* L	1
Oxalidaceae	*Oxalis corniculata* L	1
Papaveraceae	*Papaver somniferum* L	32
Physalacriaceae	*Armillariella mellea* (Vahl) P. Kumm	93
Phytolaccaceae	*Phytolacca americana* L	12
Pinaceae	*Abies nordmanniana* (Steven) Spach	7
Pinaceae	*Cedrus* sp.	3
Pinaceae	*Picea orientalis* (L.) Peterm	17
Pinaceae	*Pinus kochiana* Klotzsch ex K. Koch	10
Pinaceae	*Pinus sosnowskyi* Nakai	8
Piperaceae	*Piper nigrum* L	4
Plantaginaceae	*Plantago major* L	2
Plantaginaceae	*Valeriana officinalis* L	1
Pleurotaceae	*Pleurotus cornicopiae* (Paulet) Rolland	4
Pleurotaceae	*Pleurotus ostreatus* (Jacq. ex Fr.) P. Kumm	90
Pluteaceae	*Pluteus cervinis* (Schaeffer ex Fr). P. Kumm	28
Poaceae	*Avena sativa* L	42
Poaceae	*Bambusa* sp.	4
Poaceae	*Hordeum vulgare* L	97
Poaceae	*Hordeum vulgare* L. ssp. *vulgare* L. var. *coelestre* L	5
Poaceae	*Panicum crus-calli* L	2
Poaceae	*Panicum milanjianum* Rendle	38
Poaceae	*Secale cereale* L	65
Poaceae	*Setaria italica* (L.) P. Beauv	16
Poaceae	*Sorghum bicolor* (L.) Moench	2
Poaceae	*Triticum aestivum* L	144
Poaceae	*Triticum carthlicum* Nevski	4
Poaceae	*Triticum dicoccum* Schrank	2
Poaceae	*Triticum* sp.	2
Poaceae	*Zea mays* L	195
Polygonaceae	*Fagopyrum tataricum* (L.) Gaertn	9
Polygonaceae	*Polygonum alpinum* All	57
Polygonaceae	*Polygonum aviculare* L	9
Polygonaceae	*Polygonum carneum* C. Koch	74
Polygonaceae	*Polygonum panjutini* Kharkev	5
Polygonaceae	*Polygonum* sp.	6
Polygonaceae	*Rheum rhabarbarum* L	3
Polygonaceae	*Rumex acetosa* L	77
Polygonaceae	*Rumex acetosella* L	19
Polygonaceae	*Rumex alpinus* L	84
Polygonaceae	*Rumex crispus* L	44
Polygonaceae	*Rumex scutatus* L	6
Polygonaceae	*Rumex* sp.	20
Polygonaceae	*Rumex tuberosus* L	1
Polypodiaceae	*Polypodium vulgare* L	10
Polyporaceae	*Polyporus squamosus* (Huds.) Fr	9
Portulacaceae	*Portulaca oleracea* L	85
Primulaceae	*Cyclamen vernum* Sweet	5
Primulaceae	*Primula luteola* Rupr	1
Primulaceae	*Primula macrocalyx* Bunge	24
Primulaceae	*Primula* sp.	4
Primulaceae	*Primula vulgaris* subsp. *rubra* (Sm.) Arcang	3
Primulaceae	*Primula woronowii* Losinsk	18
Psathyrellaceae	*Coprinopsis atramentaria* (Bull.) Redhead, Vilgalys & Moncalvo	24
Ramariaceae	*Ramaria flava* (Schaeff.) Quél	18
Ranunculaceae	*Adonis aestivalis* L	2
Ranunculaceae	*Clematis vitalba* L	11
Ranunculaceae	*Ranunculus repens* L	2
Rhamnaceae	*Rhamnus imeretina* Booth, Petz. & Kirchn	1
Rhamnaceae	*Ziziphus jujuba* Mill	2
Rhododendraceae	*Rhododendron caucasicum* Pall	79
Rhododendraceae	*Rhododendron luteum* Sweet	15
Rhododendraceae	*Rhododendron ponticum* L	27
Rosaceae	*Armeniaca vulgaris* Lam	2
Rosaceae	*Aruncus vulgaris* Raf	31
Rosaceae	*Cornus mas* L	135
Rosaceae	*Cotoneaster multiflorus* Bunge	4
Rosaceae	*Crataegus curvisepala* Lindm	34
Rosaceae	*Crataegus pentagyna* Waldst	48
Rosaceae	*Crataegus* sp.	13
Rosaceae	*Cydonia oblonga* L	80
Rosaceae	*Duchesnea indica* (Andrews) Teschem	6
Rosaceae	*Eriobotrya japonica* (Thunb.) Lindl	27
Rosaceae	*Fragaria vesca* L	74
Rosaceae	*Fragaria vesca* L. "Alibaba"	1
Rosaceae	*Fragaria virginiana* Mill	12
Rosaceae	*Fragaria* x *ananassana* Duchesne ex Rozier	35
Rosaceae	*Malus orientalis* Uglizk	685
Rosaceae	*Malus pumila* Mill. var. *paradisiaca* C.K. Schneid	3
Rosaceae	*Mespilus germanica* L	81
Rosaceae	*Padus racemosa* (Lam.) Gilib	27
Rosaceae	*Prunus amygdalus* Batsch	1
Rosaceae	*Prunus armeniaca* L	30
Rosaceae	*Prunus avium* (L.) L	187
Rosaceae	*Prunus cerasus* L	78
Rosaceae	*Prunus divaricata* Ledeb	282
Rosaceae	*Prunus insititia* L	62
Rosaceae	*Prunus laurocerasus* L	63
Rosaceae	*Prunus padus* L	2
Rosaceae	*Prunus persica* (L.) Batsch	74
Rosaceae	*Prunus* sp.	33
Rosaceae	*Prunus spinosa* L	41
Rosaceae	*Prunus vachuschtii* Bregaze	20
Rosaceae	*Prunus vulgaris* Mill	4
Rosaceae	*Prunus* x *domestica* L	296
Rosaceae	*Pyracantha coccinea* M. Roem	3
Rosaceae	*Pyrus caucasica* Fed	232
Rosaceae	*Pyrus communis* L	628
Rosaceae	*Rosa canina* L	11
Rosaceae	*Rosa pimpinellifolia* Boiss	13
Rosaceae	*Rosa* sp.	140
Rosaceae	*Rubus caesius* L	27
Rosaceae	*Rubus fruticosus* L	104
Rosaceae	*Rubus idaeus* L	268
Rosaceae	*Rubus saxatilis* L	19
Rosaceae	*Rubus* sp.	60
Rosaceae	*Sorbus aucuparia* K. Koch	18
Rosaceae	*Sorbus boissieri* C.K. Schneid	2
Rosaceae	*Sorbus caucasigena* Kom	57
Rosaceae	*Sorbus torminalis* C.Crantz	20
Rubiaceae	*Coffea arabica* L	1
Russulaceae	*Lactarius deliciosus* (L. ex Fr.) S.F. Grey	31
Russulaceae	*Lactarius piperatus* (L.) Pers	27
Russulaceae	*Lactifluus piperatus* (L.) Roussel	18
Russulaceae	*Lactifluus volemus* (Fr.) Kuntze	14
Russulaceae	*Russula adusta* Pers. Fr	6
Russulaceae	*Russula emetica* (Schaeff.) Pers	6
Russulaceae	*Russula rosea* Pers	23
Russulaceae	*Russula virescens* (Schaeff.) Fr	2
Rutaceae	*Citrus limon* (L.) Burm. f	15
Rutaceae	*Citrus recticulata* Blanco	5
Rutaceae	*Citrus sinensis* Osbeck	8
Rutaceae	*Citrus unshiu* Marcov	4
Rutaceae	*Citrus* x *paradisi* Macfad	2
Salicaceae	*Salix caprea* L	1
Sapindaceae	*Acer pseudoplatanus* L	2
Smilacaceae	*Smilax excelsa* L	91
Solanaceae	*Capsicum annuum* L	204
Solanaceae	*Capsicum annuum* L. "Sweet Bulgarian"	100
Solanaceae	*Lycopersicum esculentum* L	316
Solanaceae	*Physalis alkekengi* L	7
Solanaceae	*Solanum melogena* L	63
Solanaceae	*Solanum pseudocapsicum* L	2
Solanaceae	*Solanum tuberosum* L	347
Sparassidaceae	*Sparassis crispa* Wulfen	6
Staphyleaceae	*Staphylea colchica* Steven	116
Strophariaceae	*Hypholoma fasciculare* (Huds.) P. Kumm	6
Suillaceae	*Suillus granulatus* (L.) Roussel	14
Suillaceae	*Suillus luteus* (L.) Roussel	17
Taxaceae	*Taxus baccata* L	12
Theaceae	*Camelia sinensis* L	2
Tricholomataceae	*Lepista sordida* (Schumach.) Singer	18
Tricholomataceae	*Tricholoma aurantium* (Schaeff.) Ricken	1
Tricholomataceae	*Tricholoma portentosum* (Fr.) Quél	17
Tropaeolaceae	*Tropaeolum majus* L	1
Ulmaceae	*Ulmus glabra* Huds	3
Unidentified	Unidentified species	153
Urticaceae	*Urtica dioica* L	289
Violaceae	Viola arvensis L	1
Violaceae	*Viola* sp.	41
Vitaceae	*Vitis labrusca* L	26
Vitaceae	*Vitis sylvestris* W. Bartram	2
Vitaceae	*Vitis vinifera* L	538
Zingiberaceae	*Elatteria cardamomum* (L.) Maton	4

**Table 6:** Distribution of mentions in plant families between garden and wild plants

Families	Garden	Wild	Families	Garden	Wild
Actinidiaceae	28	0	Liliaceae	6	39
Adoxaceae	6	128	Linaceae	0	1
Agaricaceae	6	225	Lythraceae	19	13
Amanitaceae	0	16	Malvaceae	14	157
Amaranthaceae	497	350	Marasmiaceae	0	12
Amaryllidaceae	853	302	Melanthiaceae	0	5
Annonaceae	1	0	Moraceae	237	11
Apiaceae	1422	490	Morchellaceae	0	13
Araceae	10	19	Musaceae	3	0
Araliaceae	1	0	Myrtaceae	11	0
Asparagaceae	7	52	Oleaceae	0	7
Asteraceae	492	252	Onagraceae	0	1
Auriculariaceae	0	10	Onocleaceae	4	31
Bankeraceae	0	10	Orobanchaceae	0	5
Begoniaceae	10	0	Oxalidaceae	2	1
Berberidaceae	10	42	Papaveraceae	4	28
Betulaceae	81	127	Physalacriaceae	0	93
Boletaceae	0	21	Phytolaccaceae	0	12
Boraginaceae	2	20	Pinaceae	3	44
Brassicaceae	899	99	Plantaginaceae	1	2
Campanulaceae	1	110	Pleurotaceae	2	92
Cannabaceae	39	13	Pluteaceae	0	28
Cantharellaceae	0	36	Poaceae	609	9
Caprifoliaceae	0	3	Polygonaceae	29	385
Caryophyllaceae	7	50	Polypodiaceae	0	10
Clavariadelphaceae	0	5	Polyporaceae	0	9
Convolvulaceae	15	2	Portulacaceae	6	79
Cornaceae	22	117	Primulaceae	0	55
Cortinariaceae	0	1	Psathyrellaceae	0	24
Corylaceae	1	3	Ramariaceae	0	12
Crassulaceae	0	32	Ranunculaceae	5	22
Cucurbitaceae	662	3	Rhamnaceae	1	2
Cupressaceae	0	2	Rhododendraceae	1	120
Dipsacaceae	0	1	Rosaceae	2683	1249
Dryopteridaceae	0	35	Rubiaceae	1	0
Ebenaceae	53	10	Russulaceae	3	124
Elaeagnaceae	1	9	Rutaceae	34	0
Ericaceae	4	472	Salicaceae	0	1
Euphorbiaceae	1	0	Sapindaceae	0	2
Fabaceae	738	101	Smilacaceae	0	91
Fagaceae	11	128	Solanaceae	1020	19
Fistulinaceae	0	6	Sparassidaceae	0	6
Fungi	2	225	Staphyleaceae	29	87
Gentianaceae	0	1	Strophariaceae	0	6
Geraniaceae	0	13	Suillaceae	0	31
Gomphaceae	0	6	Taxaceae	0	12
Grossulariaceae	226	99	Theaceae	2	0
Guttiferae	1	11	Tricholomataceae	0	36
Hericiaceae	0	1	Tropaeolaceae	1	0
Indet	24	126	Ulmaceae	0	3
Iridaceae	9	0	Urticaceae	31	258
Juglandaceae	222	27	Violaceae	0	42
Lamiaceae	550	403	Vitaceae	553	8
Lauraceae	23	4	Zingiberaceae	4	0
Lepiotaceae	0	24

**Table 7:** Pairwise comparisons with FDR *p*-value adjustment method of plant family usage between regions after significant PERMANOVA analysis (Table Permanova)

	Adjara	Guria	Javakheti Plateau	Kakheti	Khevsureti	Kvemo Kartli	Kvemo Racha	Kvemo Svaneti	Lechkhumi	Meskheti	Mtianeti	Samegrelo	Tori	Tusheti	Zemo Imereti	Zemo Racha
Guria	0.0019															
Javakheti Plateau	0.0019	0.0031														
Kakheti	0.0019	0.0019	0.0159													
Khevsureti	0.0019	0.0019	0.0044	0.0019												
Kvemo Kartli	0.0019	0.0072	0.0019	0.0370	0.0031											
Kvemo Racha	0.0117	0.0362	0.0019	0.0019	0.0019	0.0019										
Kvemo Svaneti	0.0209	0.0031	0.0019	0.0044	0.0019	0.0019	0.0044									
Lechkhumi	0.0608	0.0031	0.0019	0.0019	0.0019	0.0019	0.0019	0.0019								
Meskheti	0.0209	0.0378	0.0019	0.0031	0.0031	0.0082	0.0159	0.0126	0.0019							
Mtianeti	0.0290	0.1400	0.0019	0.0290	0.0044	0.0544	0.0209	0.0095	0.0019	0.1068						
Samegrelo	0.0019	0.0019	0.0019	0.0019	0.0019	0.0019	0.0019	0.0019	0.0019	0.0019	0.0019					
Tori	0.0107	0.0229	0.0019	0.0019	0.0019	0.0031	0.0117	0.0031	0.0019	0.0393	0.0107	0.0019				
Tusheti	0.0019	0.0019	0.0031	0.0019	0.0019	0.0019	0.0019	0.0019	0.0019	0.0019	0.0031	0.0019	0.0019			
Zemo Imereti	0.0031	0.0685	0.0019	0.0290	0.0019	0.0058	0.0019	0.0019	0.0019	0.0126	0.0082	0.0019	0.0019	0.0019		
Zemo Racha	0.0044	0.0710	0.0082	0.0229	0.0019	0.0386	0.0159	0.0019	0.0019	0.0117	0.0561	0.0019	0.0031	0.0126	0.0181	
Zemo Svaneti	0.0299	0.0019	0.0019	0.0019	0.0019	0.0019	0.0058	0.0082	0.0031	0.0474	0.0181	0.0019	0.0209	0.0019	0.0031	0.0019

Analyses were based on Euclidean distance and 999 permutations

**Table 8:** Pairwise comparisons with FDR *p*-value adjustment method of plant genus usage between regions after significant PERMANOVA analysis (Table Permanova)

	Adjara	Guria	Javakheti Plateau	Kakheti	Khevsureti	Kvemo Kartli	Kvemo Racha	Kvemo Svaneti	Lechkhumi	Meskheti	Mtianeti	Samegrelo	Tori	Tusheti	Zemo Imereti	Zemo Racha
Guria	0.0012															
Javakheti Plateau	0.0012	0.0012														
Kakheti	0.0012	0.0012	0.0012													
Khevsureti	0.0012	0.0012	0.0012	0.0012												
Kvemo Kartli	0.0012	0.0012	0.0012	0.0012	0.0012											
Kvemo Racha	0.0012	0.0022	0.0012	0.0012	0.0012	0.0012										
Kvemo Svaneti	0.0012	0.0012	0.0012	0.0012	0.0012	0.0012	0.0012									
Lechkhumi	0.0012	0.0065	0.0012	0.0012	0.0012	0.0012	0.0012	0.0012								
Meskheti	0.0012	0.0022	0.0012	0.0012	0.0012	0.0012	0.0012	0.0012	0.0012							
Mtianeti	0.0055	0.0670	0.0012	0.0153	0.0012	0.0022	0.0073	0.0073	0.0012	0.0264						
Samegrelo	0.0012	0.0012	0.0012	0.0012	0.0012	0.0012	0.0012	0.0012	0.0012	0.0012	0.0012					
Tori	0.0012	0.0022	0.0012	0.0012	0.0012	0.0012	0.0012	0.0012	0.0012	0.0022	0.0012	0.0012				
Tusheti	0.0012	0.0012	0.0012	0.0012	0.0012	0.0012	0.0012	0.0012	0.0012	0.0012	0.0012	0.0012	0.0012			
Zemo Imereti	0.0012	0.0073	0.0012	0.0022	0.0012	0.0012	0.0012	0.0012	0.0012	0.0012	0.0083	0.0012	0.0012	0.0012		
Zemo Racha	0.0033	0.0584	0.0012	0.0073	0.0012	0.0022	0.0065	0.0012	0.0012	0.0022	0.0103	0.0012	0.0012	0.0012	0.0033	
Zemo Svaneti	0.0012	0.0012	0.0012	0.0012	0.0012	0.0012	0.0012	0.0012	0.0012	0.0012	0.0022	0.0012	0.0012	0.0012	0.0012	0.0012

Analyses were based on Euclidean distance and 999 permutations

**Table 9:** Pairwise comparisons with FDR *p*-value adjustment method of different plant system used (root, shoot, or both) between regions after significant PERMANOVA analysis (Table Permanova)

	Adjara	Guria	Javakheti Plateau	Kakheti	Khevsureti	Kvemo Kartli	Kvemo Racha	Kvemo Svaneti	Lechkhumi	Meskheti	Mtianeti	Samegrelo	Tori	Tusheti	Zemo Imereti	Zemo Racha
Guria	0.0065															
Javakheti Plateau	0.0187	0.0038														
Kakheti	0.4754	0.0121	0.2596													
Khevsureti	0.4093	0.0038	0.0121	0.5112												
Kvemo Kartli	0.4093	0.0139	0.0865	0.9340	0.4054											
Kvemo Racha	0.0038	0.1808	0.0038	0.0139	0.0038	0.0065										
Kvemo Svaneti	0.5393	0.0038	0.0231	0.7329	0.5763	0.6930	0.0038									
Lechkhumi	0.2596	0.2546	0.0038	0.1539	0.0744	0.0544	0.0252	0.0415								
Meskheti	0.5393	0.1396	0.0038	0.3965	0.3660	0.1808	0.0065	0.2546	0.2343							
Mtianeti	0.7807	0.2720	0.0038	0.5731	0.5139	0.4038	0.0691	0.4871	0.2629	0.6245						
Samegrelo	0.0038	0.0038	0.0065	0.0209	0.0038	0.0038	0.0038	0.0038	0.0038	0.0038	0.0038					
Tori	0.0038	0.5112	0.0038	0.0038	0.0038	0.0038	0.2343	0.0038	0.0139	0.0038	0.0358	0.0038				
Tusheti	0.4054	0.0038	0.0647	0.7222	0.7025	0.6091	0.0038	0.7323	0.0375	0.2629	0.4559	0.0065	0.0038			
Zemo Imereti	0.0774	0.7439	0.0038	0.0680	0.0139	0.0340	0.2125	0.0321	0.3001	0.1104	0.2510	0.0038	0.4334	0.0163		
Zemo Racha	0.6609	0.4054	0.0038	0.5273	0.4054	0.4038	0.1247	0.4054	0.6800	0.6800	0.7444	0.0065	0.1060	0.4054	0.4054	
Zemo Svaneti	0.3660	0.1060	0.0038	0.1554	0.1396	0.1168	0.0038	0.1248	0.7108	0.6622	0.6887	0.0038	0.0095	0.1168	0.2149	0.7807

Analyses were based on Euclidean distance and 999 permutations

**Table 10:** Pairwise comparisons with FDR *p*-value adjustment method of different general plant parts used (vegetative, reproductive, or both) between regions after significant PERMANOVA analysis (Table Permanova). Analyses were based on Euclidean distance and 999 permutations

	Adjara	Guria	Javakheti Plateau	Kakheti	Khevsureti	Kvemo Kartli	Kvemo Racha	Kvemo Svaneti	Lechkhumi	Meskheti	Mtianeti	Samegrelo	Tori	Tusheti	Zemo Imereti	Zemo Racha
Guria	0.0020															
Javakheti Plateau	0.0020	0.0054														
Kakheti	0.0020	0.0086	0.4630													
Khevsureti	0.0020	0.0115	0.0115	0.3372												
Kvemo Kartli	0.0020	0.0071	0.6074	0.6437	0.1026											
Kvemo Racha	0.0020	0.3166	0.0020	0.0020	0.0020	0.0020										
Kvemo Svaneti	0.6074	0.0071	0.0020	0.0101	0.0020	0.0020	0.0020									
Lechkhumi	0.0020	0.0054	0.0020	0.0020	0.0020	0.0020	0.0020	0.0020								
Meskheti	0.0302	0.3671	0.0020	0.1709	0.1593	0.0158	0.0158	0.0517	0.0020							
Mtianeti	0.0915	0.4792	0.0020	0.5124	0.6437	0.1560	0.0666	0.0915	0.0020	0.7760						
Samegrelo	0.0020	0.0020	0.0020	0.0020	0.0020	0.0020	0.0020	0.0020	0.0020	0.0020	0.0020					
Tori	0.0020	0.1593	0.0020	0.0020	0.0020	0.0020	0.4439	0.0020	0.0020	0.0020	0.0038	0.0020				
Tusheti	0.0020	0.0038	0.1885	0.3411	0.0857	0.5533	0.0020	0.0020	0.0020	0.0130	0.1676	0.0020	0.0020			
Zemo Imereti	0.0020	0.5440	0.0038	0.0783	0.0260	0.0558	0.0920	0.0020	0.0020	0.0915	0.1916	0.0020	0.0020	0.0508		
Zemo Racha	0.0020	0.2997	0.0526	0.3309	0.0915	0.2964	0.0535	0.0054	0.0020	0.0581	0.1511	0.0020	0.0020	0.4792	0.3992	
Zemo Svaneti	0.2802	0.0260	0.0020	0.0020	0.0020	0.0020	0.0086	0.1119	0.0020	0.0250	0.0645	0.0020	0.0101	0.0020	0.0038	0.0020

**Table 11:** Pairwise comparisons with FDR *p*-value adjustment method of specific plant parts used (bark, branches, buds, bulb, cones, flowers, fruit, latex, leaves, resin, roots, seeds, shoots, silk, stem, timber, tuber, whole plant) between regions after significant PERMANOVA analysis (Table Permanova)

	Adjara	Guria	Javakheti Plateau	Kakheti	Khevsureti	Kvemo Kartli	Kvemo Racha	Kvemo Svaneti	Lechkhumi	Meskheti	Mtianeti	Samegrelo	Tori	Tusheti	Zemo Imereti	Zemo Racha
Guria	0.0018															
Javakheti Plateau	0.0018	0.0018														
Kakheti	0.0018	0.0018	0.0267													
Khevsureti	0.0018	0.0018	0.0033	0.0697												
Kvemo Kartli	0.0018	0.0033	0.0057	0.3999	0.0057											
Kvemo Racha	0.0018	0.1692	0.0018	0.0018	0.0018	0.0018										
Kvemo Svaneti	0.2045	0.0018	0.0018	0.0018	0.0018	0.0018	0.0018									
Lechkhumi	0.0057	0.0057	0.0018	0.0018	0.0018	0.0018	0.0018	0.0046								
Meskheti	0.0046	0.1608	0.0018	0.0603	0.0018	0.0018	0.0046	0.0173	0.0018							
Mtianeti	0.0267	0.3522	0.0018	0.3078	0.0096	0.0057	0.0324	0.0537	0.0018	0.6410						
Samegrelo	0.0018	0.0018	0.0018	0.0018	0.0018	0.0018	0.0018	0.0018	0.0018	0.0018	0.0018					
Tori	0.0018	0.0355	0.0018	0.0018	0.0018	0.0018	0.1349	0.0018	0.0033	0.0033	0.0046	0.0018				
Tusheti	0.0018	0.0018	0.0148	0.0633	0.0433	0.0714	0.0018	0.0018	0.0018	0.0018	0.0071	0.0018	0.0018			
Zemo Imereti	0.0018	0.2145	0.0018	0.0870	0.0033	0.0109	0.0222	0.0018	0.0018	0.0222	0.1272	0.0018	0.0033	0.0046		
Zemo Racha	0.0018	0.1711	0.0018	0.2492	0.0083	0.1305	0.0267	0.0018	0.0018	0.0324	0.0668	0.0018	0.0018	0.0413	0.2493	
Zemo Svaneti	0.0083	0.0057	0.0018	0.0018	0.0018	0.0018	0.0018	0.0787	0.0046	0.0334	0.0668	0.0018	0.0018	0.0018	0.0018	0.0046

Analyses were based on Euclidean distance and 999 permutations
